# Synchronous virtual care in children’s health care: a scoping review

**DOI:** 10.3389/fped.2025.1610407

**Published:** 2025-11-06

**Authors:** R. T. Zulla, D. B. Nicholas, S. Sutherland, E. Cohen, K. Birnie, S. Anthony, P. Robeson, E. Selkirk, T. Killackey, V. Mohabir, J. Stinson

**Affiliations:** 1 Faculty of Social Work, Central and Northern Alberta Region, University of Calgary, Edmonton, AB, Canada; 2 Edwin S.H. Leong Centre for Healthy Children, University of Toronto, Toronto, ON, Canada; 3 Department of Anesthesiology, Cumming School of Medicine, Perioperative and Pain Medicine, University of Calgary, Calgary, AB, Canada; 4 Child Health Evaluative Services, Hospital for Sick Children, Toronto, ON, Canada; 5 Children’s Healthcare Canada, Ottawa, ON, Canada; 6 Lawrence Bloomberg Faculty of Nursing, University of Toronto, Toronto, ON, Canada

**Keywords:** virtual care, synchronous, telehealth, pediatrics, scoping review

## Abstract

**Objective:**

Synchronous virtual care comprises real-time, online-mediated healthcare. This approach has increasingly been used in pediatrics, largely implemented in the COVID-19 pandemic. Evidence is limited on the impacts of this mode of care delivery on patient and family experience and care quality. To our knowledge, this is the first scoping review to amalgamate existing knowledge about the perceived impact of synchronous virtual care as it is experienced by children and their families across multiple disciplines.

**Methods:**

Following guidance from the Joanna Briggs Institute, a search of the peer reviewed, published literature was conducted employing multiple databases: APA PsycInfo, CINAHL, EBSCO, Embase, and OVID. Reviewed articles were published in English from January 1, 2013 to December 31, 2023, and addressed virtual care for children and their families. The initial search generated 1,079 articles, which underwent abstract and then full-text screening. A total of 157 full text articles were screened, yielding 117 articles from which data was extracted.

**Results:**

Virtual care interventions, generally appearing in the last decade (2013–2023), have been largely studied using quantitative approaches. They tend to be positively viewed by youth and parents as indicated by identified benefits and general satisfaction. However, articles report both facilitating and hindering elements of virtual care, and barriers are reported that reflect inequities associated with social determinants of health. Such barriers are shown to impede the use of virtual care among some marginalized communities. The review indicates that effective virtual care approaches require (a) program/organizational infrastructure support, (b) training for both service providers and users, and (c) tailoring to clinical needs.

**Conclusion:**

Considering virtual care “fit” for target patients and families is important. Implications for clinical care as well as guidelines for future research are offered.

## 1 Introduction

Virtual care is an umbrella term for technology-based support in healthcare. Diverse terms to describe virtual care or telehealth have been used such as synchronous monitoring (1–3), telerehabilitation (4), eHealth (1, 5), mHealth (6), and web-based education (7). Pediatric virtual care has been applied in a range of clinical populations such as children and youth with autism (2), chronic illness (5), cancer (8), and acquired brain injury (9). At present, there is a growing body of reviews assessing the role of virtual care, however, these reviews have specific focus on (i) populations with specific conditions such as children with special health care needs (1), autism (2), asthma (3), chronic pulmonary disease (5), diabetes (10) and chronic illness (11), (ii) delivery within specific pediatric specialties such as rehabilitation (4) or surgery (6), (iii) certain online platforms such as mobile, eHealth or mHealth (6–9), (iv) geographical regions such as rural settings (12) and (v) research designs such as interventions that have been evaluated using random controlled trials (13).

Reviews have examined benefits and limitations of virtual care. Overall, general acceptance of virtual care in clinical practice and research has been reported by patients and family caregivers (3, 5–9, 12–16), physicians (17), and a range of health care professionals (18–23). Reviews conclude that virtual care can reduce costs related to travel (20, 24) for family caregivers and lower staff labor costs for organizations (18). However, patients and/or their caregivers have cited challenges with virtual care including limited personal interaction (24), technological challenges (24), and concerns with security of health information (6, 16) and privacy risks (14).

During the COVID-19 pandemic, there was widespread implementation of virtual care. While the literature has variably addressed the access, use, and impact of virtual care, there has been limited attention given to patient and family experiences of virtual care, thus raising questions about children/youth and caregiver preferences in using virtual care. To address this gap, a scoping review of the last decade of literature was undertaken to assess how virtual care intervention have been delivered and evaluated, and have impacted children and their families. An additional aim was to review how interventions incorporated equity considerations, particularly the social determinants of health (SDOH). We defined SDOH as “the conditions in the environments where people are born, live, learn, work, play, worship, and age that affect a wide range of health, functioning, and quality-of-life outcomes and risks” (25). SDOH also consider the broader forces and systems that shape daily life including economic and social policies, norms, political structures and systemic factors (e.g., racism, ableism, sexism) (25). To this end, this study addressed the following questions: (1) What virtual care approaches have been used in pediatric care relative to child and family experiences and benefits, (2) What are the aims and how are virtual care approaches implemented in clinical practice?, (3) What are families’ experiences and perceptions of virtual care?, (4) What study approaches have been used to evaluate children/youth and family experiences of virtual care?, and (5) How are SDOH considered in the design and/or implementation plans of virtual care?

## 2 Methods

The scoping review was informed by guidelines outlined by the Joanna Briggs Institute (JBI) (26, 27). A search of the literature published between January 1, 2013, and December 31, 2023, was led by a research colleague with expertise in scoping reviews using APA PsycInfo, CINAHL, EBSCO, Embase, and MEDLINE electronic databases. Search terms were as follows: virtual (e.g., telemedicine, telehealth, telecare, teleconference, Zoom), pediatrics (e.g., infants, child, toddler, adolescent, youth), hospital (e.g., secondary care, tertiary care, inpatient, outpatient) and experience (e.g., perspectives, satisfaction, preferences) (search strategy in Table 1). Study inclusion criteria were: (1) peer-reviewed, (2) published in English, (3) focused on pediatric synchronous virtual care (e.g., online and/or phone) based on the following definition, “.real-time, virtual, direct-to-patient appointments. Synchronous telehealth happens in live, real-time settings, in which the patient interacts with a provider(s), usually via phone or video. Providers and patients communicate directly, often resulting in a diagnosis, treatment plan, and/or prescription” (28), (5) inclusive of pediatric patients (including birth to 18 years) and/or their family, (6) inclusive of primary data (e.g., surveys, interviews), and (7) inclusive of outcomes of family satisfaction, preferences and/or perspectives about the virtual care approaches. Exclusion criteria consisted of: (1) conference/meeting, proceedings, congress, guidelines, dissertation, or a review (e.g., literature review, systematic review, scoping review, meta-analysis), (2) sole focus on healthcare provider perspectives, (3) only administrative data provided, (4) development of a test or model of virtual care, and/or (5) focus on obstetrical care. At the last stage, when we extracted information from a full text review of each article, we examined how SDOH were addressed, including equity considerations (29, 30). Addressing these considerations emerged as critical in bringing an equity, diversity, inclusion, and accessibility (EDIA) lens to this work, in terms of interrogating study inclusion of diverse groups relative to the utility, impact and experience of virtual care. For reviews such as this current one, an EDIA lens can highlight how marginalized groups may experience unique challenges in accessing care.

**Table 1:** Search strategy by database.

Database	Search strategy and terms
OVID database including the following database: EMBASE APA PsycInfo OVID medline	Search #1: Explore the term, telemedicine/
	Search #2: (telehealth or telemedicine or telecare or telepsych* or teleconsult* or teleconferenc* or video?conferenc* or Zoom or Skype or “text messag*” or SMS).kf,tw.
	Search #3: [(virtual or remote or digital or mobile or online) adj3 (care or deliver* or appointment* or consult* or intervention* or monitor* or test* or diagnos*)].kf,tw.
	Search #4: Combine records using terms from Search #1-3
	Search #5: Explore the term, Child Health Services/
	Search #6: Explore the term, Pediatrics
	Search #7: (p?ediatric* or infan* or baby or babies or newborn* or neonat* or perinat* or child* or toddler* or juvenile or adolescent* or youth or teen*).kf,tw.
	Search #8: (child* or adolesc* or pediat* or paediat* or infan* or newborn* or neonat* or perinat*).jn.
	Search #9: Combine records using terms from Search #5–7
	Search #10: Explore the term, Hospitals/ or Inpatients/ or Outpatients/
	Search #11: (“hospital*” or “secondary care” or “tertiary care” or “quaternary care” or inpatient or outpatient). kf,tw.
	Search #12: Combine Search #10 or 11
	Search #13: (experien* or perspective* or “patient satisfaction” or “patient preference” or “patient-reported outcome measures”).kf,tw.
	Search #14: Look for terms from Search #4, 9, 12 and 13
	Search #15: Limit Search #14 to 2013 – Current
	Search #16: Look for terms from Search #14 and 15
	Search #17: Limit to Search # 16 to (books or chapter or conference abstract or conference paper or “conference review” or editorial or letter or “review” or comment or lecture or meta analysis or newspaper article or “systematic review”)
	Search #18: Exclude records from Search# 16 not 17
	Search #19: Limit to Search #18 to English
	Search #20: Combine Search #18 and 19
	Search #21: From Search #20 keep records 1–1,520
EBSCO & CINAHL	Search #1: AB (telehealth or telemedicine or telecare or telepsych* or teleconsult* or teleconferenc* or video?conferenc* or Zoom or Skype or “text messag*” or SMS) OR TI (telehealth or telemedicine or telecare or telepsych* or teleconsult* or teleconferenc* or video?conferenc* or Zoom or Skype or “text messag*” or SMS) OR MH (telehealth or telemedicine or telecare or telepsych* or teleconsult* or teleconferenc* or video?conferenc* or Zoom or Skype or “text messag*” or SMS) OR AB [(virtual or remote or digital or mobile or online) N3 (care or deliver* or appointment* or consult* or intervention* or monitor* or test* or diagnos*)] OR TI [(virtual or remote or digital or mobile or online) N3 (care or deliver* or appointment* or consult* or intervention* or monitor* or test* or diagnos*)] OR MH [(virtual or remote or digital or mobile or online) N3 (care or deliver* or appointment* or consult* or intervention* or monitor* or test* or diagnos*)]
	Search #2: AB (pediatric* or paediatric* or infan* or baby or babies or newborn* or neonat* or perinat* or child* or toddler* or juvenile or adolescent* or youth or teen*) OR TI (pediatric* or paediatric* or infan* or baby or babies or newborn* or neonat* or perinat* or child* or toddler* or juvenile or adolescent* or youth or teen*) OR MH (pediatric* or paediatric* or infan* or baby or babies or newborn* or neonat* or perinat* or child* or toddler* or juvenile or adolescent* or youth or teen*)
	Search #3: AB (hospital* or “secondary care” or “tertiary care” or “quaternary care” or inpatient or outpatient) OR TI (hospital* or “secondary care” or “tertiary care” or “quaternary care” or inpatient or outpatient) OR MH (hospital* or “secondary care” or “tertiary care” or “quaternary care” or inpatient or outpatient)
	Search #4: AB (experien* or perspective* or “patient satisfaction” or “patient preference” or “patient-reported outcome measures”) OR TI (experien* or perspective* or “patient satisfaction” or “patient preference” or “patient-reported outcome measures”) OR MH (experien* or perspective* or “patient satisfaction” or “patient preference” or “patient-reported outcome measures”)
	Filters: English, peer-reviewed journals, 2013-current

**Figure 1:**
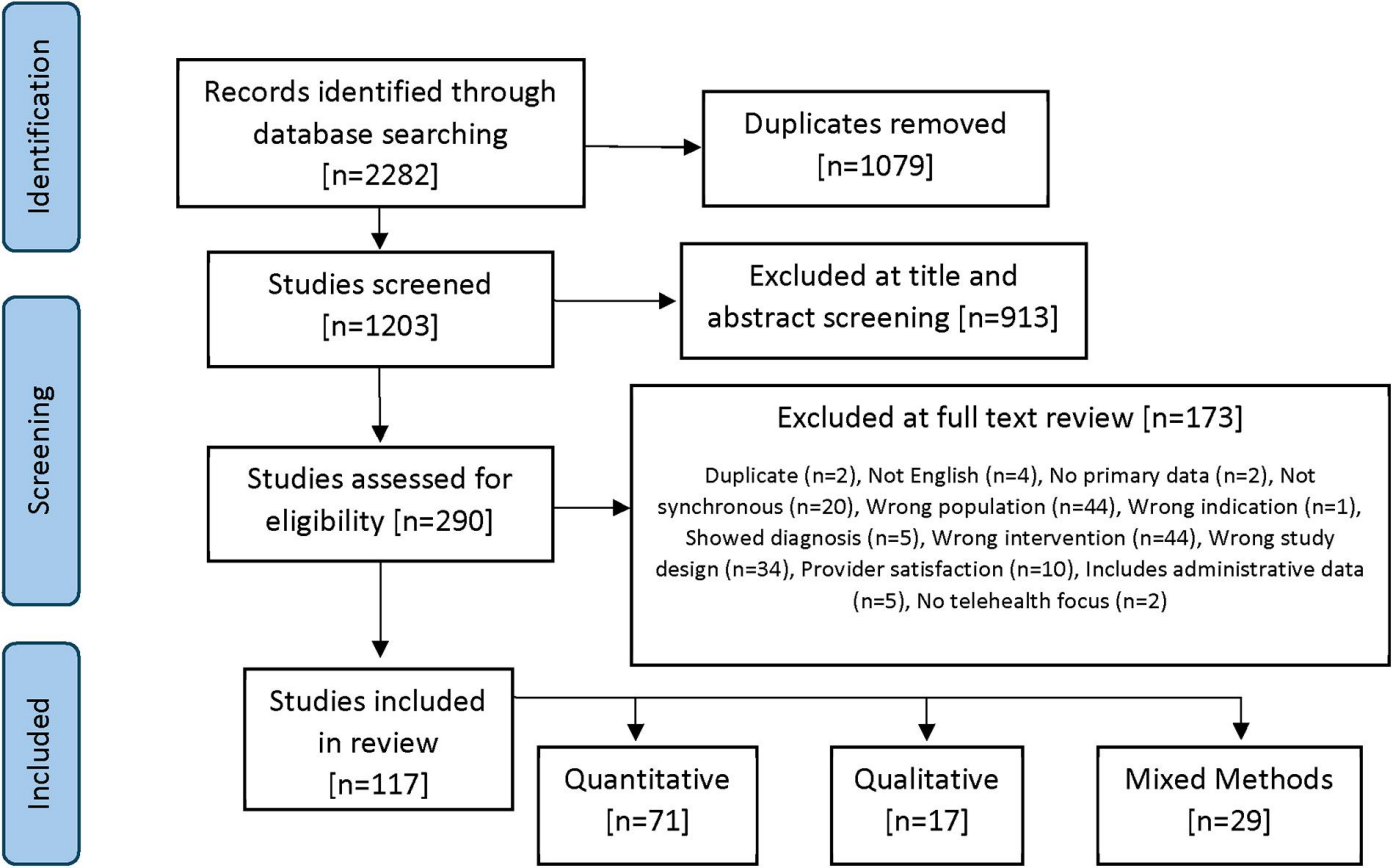
PRISMA flow diagram of the scoping review process.

Using Covidence Systematic Review software, the search yielded 1,079 articles after removal of duplicates. Two rounds of screening were conducted, as follows. Two independent reviewers examined all titles and abstracts, and a third reviewer resolved disagreements as needed. Based on inclusion and exclusion criteria, the number of included articles was reduced to 157, which were subsequently advanced for full article review. This second review resulted in 117 articles (Figure 1). Article review was conducted by team leads (RTZ, SS), with supervision by team members who bring extensive experience in secondary review. As this was a scoping review, institutional ethics board approval was not required.

### 2.1 Data extraction

Following full-text review, a total of 117 articles, as noted above, were included in this review. Through an iterative process, we generated a table to document specific features of existing studies. These articles were independently coded for retrieval of the following information: study/article title, year of publication, author(s), country, study objectives, research questions/hypothesis, study design, data collection methodology, sample size, sample characteristics, purpose of study, definition of virtual care approach, subspecialty in which approach was studied, how approach was implemented, analysis methods, outcomes, key recommendations, and study strengths and limitations. Two reviewers (RTZ, SS) extracted and analyzed the data. Given that this initiative was a scoping review, we did not appraise study design methods.

## 3 Results

We addressed key questions to interrogate virtual care in reviewed articles, as follows: (1) how has virtual care been implemented?; (2) what are study objectives and aims of the virtual care application?, (3) by whom, when, where and how has virtual care been evaluated?, (4) how does virtual care address SDOH?, and (5) how do children, youth and families experience virtual care (e.g., benefits, satisfaction, challenges, and advice offered in improving care)? These questions are addressed below. Within the Supplementary Section, Supplementary Table 1 provides the full list of the 117 articles including details of each study aim and design, the virtual care intervention addressed and outcomes. Supplementary Table 2 summarizes findings reported in individual studies.

### 3.1 How has virtual care been implemented?

Virtual care approaches varied in terms of how they were described and the modalities, devices or software used to connect patients/families with their healthcare provider. Of the 117 articles analyzed, 56 (48%) used videoconferencing as their mode of communication; however, the approach description and its purpose varied, as outlined in Table 2.

**Table 2:** Number of articles per virtual care/telehealth approach.

Platform for virtual care/telehealth approach	Research approach		
	Quantitative	Qualitative	Mixed Methods
	Number (%)	Number (%)	Number (%)
Video	34 (29)	8 (7)	14 (12)
Multiple methods (e.g., telephone, video, text, and/or email)	13 (11)	–	4 (3)
Telephone	14 (12)	–	3 (2)
Video or telephone	–	1 (0.8)	1 (0.8)
Hybrid (e.g., telephone + in-person)	11 (9)	4 (3)	4 (3)
No information reported	2 (1)	1 (0.8)	3 (2)
No intervention given (^a^Note: Articles only asked caregivers or youth perceptions of telehealth)	3 (2)	3 (2)	–

a More than one platform could be recorded for each article in this table.

Many articles (*n* = 14; 12%) used a range of approaches from which patients and families could choose (e.g., videoconferencing or telephone calls), based on what worked best for the child and family. In some articles (*n* = 17, 15%), parents/caregivers were required to gather and upload child/patient information to an online portal or send it via email pre/post appointment. For instance, a telemedicine cardiac program offered education and support through telephone mediation and required families to upload their child's health data via an app prior to a clinical visit (31). Some articles (*n* = 17, 15%) contrasted modalities (e.g., in-person vs. virtual) to determine if virtual care was a viable option based on satisfaction and/or care outcomes.

### 3.2 What are study objectives and the aims of the virtual care applications?

All reviewed articles focused on virtual care; however, specific aims varied. Most commonly, articles explored experiences of, or level of satisfaction with, virtual care (*n* = 47; 40%), followed by articles evaluating satisfaction with virtual care particularly during the COVID-19 pandemic (*n* = 36, 31%). Not surprisingly, there was a notable increase in papers focusing on virtual care during the height of the pandemic, i.e., the pandemic was determined to be between March 2020 and May 2023 (32).

A common aim across articles was to contrast virtual care with face-to-face care approaches (*n* = 20, 17%). In some articles (*n* = 19; 16%), virtual care was developed to seek continuity of care and/or offer care during the COVID-19 pandemic. Varied study objectives and virtual care approach aims are outlined in Table 3, with several articles having multiple aims.

**Table 3:** Distribution by purpose of study and virtual approach^a^.

Study objectives	Number of articles (*n*)			
	Quantitative	Qualitative	Mixed methods	Total
To explore experience and/or satisfaction with virtual care	17	11	19	47
To explore experience and/or satisfaction with virtual care during the COVID-19 pandemic	25	4	7	36
To compare virtual care with face-to-face consultations	14	–	6	20
To explore the potential for virtual care intervention	–	2	–	2
Virtual care approach aims				
To improve access to care	9	6	7	22
To provide care during the COVID-19 pandemic	9	4	6	19
To explore the effectiveness of virtual care to monitor patient care/wellness	5	–	–	6
To improve care or care management	6	4	10	20
To conduct an assessment	5	–	2	7
To improve communication between families and providers	–	2	2	4
Other, unclear, or unknown	1	1	2	4

a More than one purpose could be selected per article.

### 3.3 By whom, when, where and how has virtual care been evaluated?

As shown in Table 4, articles were led by varying disciplines and specialty areas. Virtual care experiences were largely gathered from family caregivers solely (*n* = 47, 40%), from both family caregivers and children/youth (*n* = 57, 49%), and from children/youth solely (*n* = 15,13%). Most articles were published after 2019 (2 articles in 2013, 3 in 2015, 1 in 2016, 2 in 2017, 3 in 2018, 6 in 2019, 23 in 2020, 25 in 2021, 32 in 2022, and 20 in 2023). The number of articles remained relatively stable from 2013 to 2019, with an average of 3 per year. However, from 2020 to 2023 this number exponentially rose to a mean of just under 25 per year. This rapid rise likely reflects the limited in-person care available during the COVID-19 pandemic; hence, heightened attention devoted to virtual care as a common care offering and requisite. Notably, 63% of the total articles included quantitative approaches (33–75) that drew on data collected during the COVID-19 pandemic (based on pandemic dates of March 2020 to May 2023 (32), as noted above.

**Table 4:** Number of articles per specialty area and study type (*n* = 117).

Specialty	Quantitative	Qualitative	Mixed methods	Total
Multiple specialties (e.g., sample from across hospital rather than within specific department)	7	3	6	16
Surgical specialties				
General surgery	6	2	1	9
ENT	7	–	–	7
Cardiothoracic	5	–	1	6
Urology	2	–	1	3
Orthopedics	1	–	1	2
Ophthalmology	2	–	–	2
Dentistry	1	–	1	2
Transplant	–	–	1	1
Pediatric subspecialties				
Neonatal intensive care unit	5	4	3	12
Endocrinology	5	1	3	9
General pediatric	6	1	1	8
Emergency medicine	4	1	1	6
Gastroenterology	3	1	1	5
Neurology	3	1	–	4
Chronic pain	–	–	2	2
Critical care	–	1	–	1
Dermatology	1	–	–	1
Oncology	1	–	–	1
Rheumatology	1	–	–	1
Osteopathy	1	–	–	1
Neurodevelopmental and mental health				
Psychiatry/Psychology	5	2	3	10
Rehabilitation^a^	4	–	2	6
Neurodevelopmental	1	–	1	2

a Physical, speech, occupational, cognitive-behavioural, neuropsychological, psychological support.

Articles were conducted in various world regions; however, almost half were based in the United States (*n* = 52, 44%; Table 5). Methodologies varied across articles. Quantitative articles tended to evaluate virtual care, using a range of approaches including observational design (cohort/case study/cross-sectional/prospective/longitudinal) (*n* = 38, 54%) (33–35, 38–41, 44, 47, 48, 53, 54, 56, 58–60, 62–66, 68, 70–72, 76–87), exploratory design (*n* = 19, 27%) (37, 42, 45, 49, 50, 55, 61, 69, 88–99), and experimental design (*n* = 11, 15%) (36, 49, 51, 87, 100–103) [Table 6]. Methods of data collection in articles almost exclusively used surveys (*n* = 64,90%) (33–44, 46–48, 51, 53–57, 59, 61–66, 68–70, 72–74, 77–79, 81–85, 88, 89, 91, 92, 95, 97, 98, 100–106), measured with Likert scales and/or dichotomous responses, although observation data (*n* = 4, 3%) (45, 60, 80, 87) were also collected (e.g., indication of improvement in health between admission and discharge via observation of cardiovascular endurance), along with mixed methods (e.g., survey and observation) (*n* = 1, 1%) (52).

**Table 5:** Distribution of included articles by country (*n* = 117).

Country published	Number of articles, *n* (%)			
	Quantitative	Qualitative	Mixed articles	Total
United States	39 (33)	4 (3)	9 (8)	52 (4)
United Kingdom	7 (6)	2 (2)	2 (2)	11 (9)
Australia	5 (4)	1 (1)	3 (3)	9 (8)
Canada	3 (3)	1 (1)	4 (3)	8 (7)
Germany	3 (3)	1 (1)	2 (2)	6 (5)
Italy	6 (5)	–	–	6 (5)
Sweden	–	2 (2)	2 (2)	4 (3)
Brazil	1 (1)	1 (1)	–	2 (2)
India	1 (1)	1 (1)	–	2 (2)
Saudi Arabia	2 (2)	–	–	2 (2)
Hong Kong	–	–	1 (1)	1 (1)
Norway	–	1 (1)	–	1 (1)
Argentina	1 (1)	–	–	1 (1)
Austria	–	–	1 (1)	1 (1)
Chile	–	–	1 (1)	1 (1)
Denmark	–	1 (1)	–	1 (1)
Dominican Republic	–	–	1 (1)	1 (1)
Egypt	–	–	1 (1)	1 (1)
France	–	1 (1)	–	1 (1)
Iran	–	1 (1)	–	1 (1)
Ireland	–	–	1 (1)	1 (1)
Israel	–	–	1 (1)	1 (1)
Jordan	1 (1)	–	–	1 (1)
Singapore	1 (1)	–	–	1 (1)
Spain	1 (1)	–	–	1 (1)

**Table 6:** Quantitative articles by research design and data collection (*n* = 71).

Research design	Number of articles, *n* (%)
Observational (cohort/case study/cross-sectional/prospective/longitudinal)	38 (54)
Exploratory	19 (27)
Experimental (pilot)	9 (12)
General or limited details on design	3 (4)
Data collection method	Number of articles, *n* (%)
Survey	64 (90)
Observation	4 (6)
Mixed data collection (interviews + observation)	1 (1)
No stated data collection	2 (3)

Qualitative articles elicited participant experiences and/or perspectives about virtual care [Table 7]. These articles utilized a range of qualitative approaches such as phenomenology (107, 108) and grounded theory (99). Two articles (109, 110) did not provide details about study design. Table 8 summarizes data collection methods in articles using mixed methods approaches.

**Table 7:** Distribution of qualitative articles by research design and data collection (*n* = 17).

Research design	Number of articles, *n* (%)
Limited details on qualitative design	8 (47)
Specific qualitative design (e.g., phenomenology, grounded theory)	4 (24)
Evaluation (e.g., quality improvement or trial evaluation)	3 (18)
No design detail	2 (12)
Data collection method	Number of articles, *n* (%)
Individual interviews	6 (35)
Focus groups	5 (29)
Approach not reported^a^	6 (35)

a Interviews were indicated, but it is not clear if these were individual or group-based.

**Table 8:** Distribution of mixed methods articles by research design and data collection (*n* = 29).

Research Design	Number of articles, *n* (%)
Mixed methods	29 (100)
Data collection method	Number of articles, *n* (%)
One type of data collection	
- Survey (free text responses + structured responses)	6 (21)
- Survey (structured responses)	3 (10)
- Survey (no details on structure of responses)	1 (11)
Two types of data collection	
- Medical chart + survey	5 (17)
- Survey + interviews	7 (24)
- User data (gathered on device) + interviews	1 (11)
- User data (gathered on device) + survey	1 (11)
Three types of data collection	
- Medical chart + survey + interviews^a^	5 (17)

^a^Two articles came from a single study, but each reported a different design (e.g., quantitative or qualitative).

### 3.4 How does virtual care address the social determinants of health?

We examined if, and if so how, articles addressed SDOH and equity considerations (Table 9). Addressing SDOH is important in considering care approach as it can be an indicator of resource/approach accessibility and affordability for families (e.g., buying a device for virtual care, affording a needed internet plan), reflecting salient requisites for virtual care such as access to the internet, level of technical proficiency, availability for care interactivity (e.g., during working hours), and barriers to accessing virtual care. In a recent review of the literature on the intersection of SDOH and telemedicine, Romain and colleagues (29) identified factors that hindered families’ access to telemedicine including not having broadband internet, inability to afford and/or maintain personal digital devices (e.g., desktop or laptop computer), poor digital literacy (e.g., not knowing how to find, process, discuss and share digital content), low English proficiency, and lack of or no internet infrastructure (e.g., poor broadband access, Internet “dead zones”) in specific regions.

**Table 9:** Inclusion/exclusion criteria for all articles based on social determinants of health (*n* = 117).

Inclusion criteria	Articles, *n* (%)	Exclusion criteria	Articles, *n*
Treated in an urban hospital	45	Inadequate access to technology	11
Ability to read and speak a specific language	18	Current mental health issues	6
Medically stable	16	Distance to clinic/hospital was outside range^b^	5
Families had their own electronic device	7	Language barriers	3
Distance to clinic/hospital was within range	3	Required to cross a border checkpoint for care	1
Habitability conditions of the home^a^	2	Incarcerated caregiver	1
Members of a specific ethnic group	1	Strictly palliative goals of care	1
		Participant has private insurance	1

a Composition of the family group, individual room for the patient has proper amenities, cleanliness condition of the home, availability of the minimum infrastructure for the patient's personal hygiene, and the ability to comply with the prescribed diet, environmental conditions of noise, and ambient temperature.

b Some articles required that families be in a geographic range for in-person appointments when needed.

Geographically, articles tended to reflect work in urban hospitals (*n* = 45, 38%), and often did not include participants living in rural or remote communities. Inclusion criteria varied regarding participants’ ability to read and speak a specific language (e.g., English or Spanish; *n* = 18, 15%). Study participants/families were variably required to have an electronic/technology device (*n* = 7, 6%), thus were rendered ineligible from study participation if lacking technology and/or technology access (*n* = 11, 9%). Such inclusion requirements systematically decrease representation of families with lower socioeconomic status and/or less access to the Internet.

### 3.5 How do families experience virtual care?

Articles reported a range of virtual care experiences and perceived outcomes. Generally, articles reported on benefits (*n* = 99, 85%) and challenges (*n* = 75, 64%), satisfaction (*n* = 62, 53%), utilization rates (*n* = 33, 28%), and approach preferences (*n* = 34, 29%). These findings are addressed below, as benefits, satisfaction, challenges, patterns of use, and recommendations.

#### 3.5.1 Benefits of virtual care

Most articles (*n* = 99; 85%) identified one or more benefits of virtual care. Reported child benefits included (a) greater access to care and treatment options, (b) improved health, and/or decreased occurrence of acute episodes (e.g., seizure frequency) (31, 78, 111–113), and (c) heightened child/youth capacity to manage their care (114) and share their perspectives (115).

Parents/caregivers reported vicarious benefits of virtual care for themselves and/or their family. For instance, parents/caregivers reported increased ability to monitor their child's health (76, 107, 111, 116–119). Greater involvement in children's healthcare was reported to result in positive psychological impacts in the parent/caregiver-child relationship (77, 117, 120–122). One study reported that parental involvement in a virtual tele-rehabilitation intervention enabled parents/caregivers to spend more time with their child, including collaborating on treatment goals (121). Parents/caregivers also reported psychological benefits such as increased confidence (84, 94), a sense of self-efficacy (e.g., sense of control) (87, 116, 119, 122, 123), improved flexibility via engaging in appointments from home, decreased distress, and improved self-regulation (40, 48, 102, 124). In one study, fathers reported greater capacity for caregiving tasks (78).

Articles also reported improved relations between parents/caregivers and their children's healthcare providers (53, 94, 99, 107, 113, 117, 121, 125). In these articles, parents reported healthcare providers valuing or respecting their perspectives (36, 51, 113, 117, 126) and being more approachable (113). Moreover, parents reported feeling included in decisions related to their child's care (107, 126).

Virtual care enabled parents/caregivers to spend less time commuting to clinical appointments, thus reducing family expenses (107, 111, 114–116, 119, 121, 123, 126–128), and as a result, families were able to spend more time together (114, 127–129) and/or more time at work/school (107). In one study, cost savings associated with virtual care ($22.47 USD/session) were noted, with a reduction in distance traveled (132 miles or 212.4 kilometers) and time taken (210 min) through virtual care appointments (83). Virtual care further offered a sense of expedited care (41, 128) and continuity of care/access. This was particularly helpful during the COVID-19 pandemic (107, 110), with parents expressing gratitude that virtual care reduced risks of COVID-19 exposure and infection (110, 113, 115, 119, 126).

#### 3.5.2 Satisfaction with virtual care

Over half of the articles (*n* = 62, 53%) focused on virtual care satisfaction, largely from parent/caregiver perspectives. Generally, satisfaction was reported in quantitative articles in that 82.1% of these studies indicated satisfaction with the care received, and 87.2% indicated satisfaction with the platform/approach used. Two articles reported unanimous family/caregivers and children/youth satisfaction with the virtual care provided (57, 63). A few quantitative articles (*n* = 35, 30%) reported increased satisfaction including improved access, experience or outcomes. For instance, one article (76) reported increased satisfaction over time, i.e., from 3 months (57%) to 12 months (84%). This change can be attributed to the initial hesitancy among families at the beginning of utilization, compared to more confidence and familiarity with the equipment supplied as well as, in this study, parents increasingly seeing wound healing/resolution.

Qualitative and mixed method articles reported satisfaction as contingent on specific aspects of the approach. For example, one article (117) reported that parents perceived video consultation to be safer, associated with a sense of closeness, and conducive to a more natural way of talking. Additionally, another (130) reported that telephone peer support supplemented in-person support in the hospital and was commonly rated by parents as helpful due to the expertise, care guidance, and emotional support received from peers.

#### 3.5.3 Challenges with virtual care: families

Articles reported challenges (*n* = 75, 64%) that were largely attributed to difficulties with technology. Almost a quarter of quantitative articles reported some degree of technology barriers/issues (*n* = 27, 23%). Qualitative and/or mixed methods articles identified virtual care application issues including challenges with a) setting up or logging in (114, 120, 121, 126, 131), b) audio/visual problems (107, 114, 115, 119, 126, 127, 131–134), c) connectivity (107, 113, 115, 116, 120, 126, 127, 133, 134), d) inoperable technical equipment (31, 107, 119, 131, 135), e) prohibitive costs (e.g., not having a reliable battery, needing to purchase specific medical equipment) (119, 128, 131, 133), f) ability and comfort in using the technology to upload/gather information about the child's health (111, 128), and g) resources (e.g., not having Internet or a data plan) (112, 121, 125). Technology challenges resulted in difficulties in care (107, 119). For instance, in two articles, the quality of images using home-based equipment (e.g., a tablet) (107, 119) was poor and thus required in-person follow-up (119).

##### 3.5.3.1 Challenges with virtual care: health care providers

Issues in implementing a virtual application emerged related to healthcare staff and organizational resources. For instance, parents/caregivers (a) experienced delayed responses from a healthcare provider (119, 131), (b) did not access the right health care provider (119), (c) lacked a contact for troubleshooting issues (74), (d) viewed healthcare consultations as overly time-consuming (119) or too complicated to understand (107), (e) reported difficulty retaining information (105), and/or (f) felt invalidated by their child's healthcare provider (120, 121).

Transitioning from in-person to virtual care required adaptation, and in some cases, resulted in relational and ethical challenges. Some parents/caregivers initially were unfamiliar with virtual platforms (97, 118, 126, 135), but as familiarity increased, they perceived virtual platforms to be comparable or better than in-person visits. However, relational challenges arose as families highlighted the loss of interactivity when using virtual care (e.g., not being able to view non-verbal cues, lack of eye contact) (108, 118, 126, 129, 133), with reported negative relational impacts (110). In some situations, care could not be delivered virtually. For instance, parents/caregivers were anxious because they could not obtain a much needed physical examination for their child due to prolonged restrictions during the pandemic (85). The lack of opportunity for physical examination was a major challenge when the attending healthcare provider needed to observe and assess the child in-person to determine next steps (39, 48, 55, 58, 62, 63, 65, 73, 75). In one study (40), parents/caregivers were asked to perform osteopathic interventions guided by a physician via video, but many reported difficulty and discomfort with this modality for such a task. This finding was echoed in another study in which parents/caregivers were provided with a pulse oximeter for home use, but they reported difficulty using this device (47). In reviewing this study, there were no details offered regarding whether the parents/caregivers received any instructional training on how to use the pulse oximeter.

Confidentiality and privacy issues also emerged in virtual care. Articles identified concerns articulating mental health symptoms via video (133), such as a lack of a visual roadmap for sharing private information with the healthcare provider (119), and the use of alternative spaces (e.g., sitting in a car) for therapy (108).

#### 3.5.4 Patterns of using virtual care

Utilization-related considerations included (a) type of medical appointment (121, 127), (b) meeting/appointment duration (31, 62, 93, 125, 127), (c) type of virtual care platform used (94, 114, 136), (d) wait times (69, 88, 92), (e) amount of time spent using the virtual care platform (35, 77, 125, 126, 131), and (f) receiving education from a provider (59, 130).

Several articles identified contextual elements that were attributed to approach use. In three articles, “no-show” rates were due to technology issues (63, 74, 95). One such study suggested that optimizing applications via patient/family reminders, translator assistance, and briefer download speeds may decrease rates of no-shows (74).

#### 3.5.6 What advice do families offer in delivering virtual care?

Several articles (*n* = 34, 29%) identified parent/caregiver and child preferences for virtual care design and implementation. Whether or not a virtual care application should be used was determined to be contingent on the platform (134), the type of care visit (e.g., follow-up) (110), the number of visits (110), familiarity with the technology (73, 119), and patient and family preference regarding how they could, and/or wished to, communicate with their healthcare provider. For instance, parents/caregivers without a strong understanding of English preferred face-to-face over virtual interactions (126).

Some articles recommended virtual care approach improvements (94, 114, 126, 131). As an example, families receiving hybrid care (in-person home care supplemented by virtual care) to support their infant at home wished for additional components to their virtual care such as a “better diagram function, direct contact with the nurse through, for example chat, compatibility with other web browsers, daily e-mail reminders to answer the questions, and more links to sites containing information about the growth and development of premature infants” (94) (p.7).

Articles reported facilitating vs. hindering factors related to virtual care. For instance, one study reported that adolescents and parents viewed the effectiveness of virtual care to be contingent on factors such as the severity of the child's condition and the nature of the child's relationship with the healthcare provider (134). In another study, virtual care was impeded as parents weren't given sufficient instructions regarding the virtual care process, and how to get in contact with their doctors (113). One study (110) noted gaps such as unreliable technology and internet connectivity; again, amplifying barriers related to SDOH and inequities in virtual care to marginalized communities.

## 4 Discussion

Virtual care approaches were conveyed as often implemented on a video-based platform, located largely within urban settings. Despite some variation of perceived impacts including reported barriers for marginalized communities, studies generally indicated benefits of implementing virtual care in pediatrics, as identified by families. Accordingly, virtual care generally was determined by children and their parents/caregivers as favorable. Findings also demonstrated that platforms and ways to implement virtual care approaches can vary relative to the needs of the child/youth and/or the parent/caregiver. These findings are complementary to results in other secondary reviews in this area which convey virtual care as acceptable, with emerging evidence of beneficial outcomes in care delivery. As an example, Shah & Badawy's (13) review of randomized controlled trials addressing telemedicine approaches demonstrated improved or comparable impacts of telemedicine compared to controls, based on outcomes of symptom management, satisfaction, quality of life, medication use adherence, visit completion, and disease progression.

This review adds to the overall literature by synthesizing primary studies specifically from the perspective of children and their families. In inclusively focusing on this literature across clinical areas and populations, this review importantly incorporates virtual care in a wide range of practice areas and methodological approaches. Other secondary reviews tend to address specific clinical areas or study designs. As an example, Mitra et al. (15) conducted a systematic review of telemedicine in pediatric emergency care, and Faical et al. (5) reviewed telehealth articles addressing youth with chronic pulmonary disease. Both reviews found overall benefits of virtual care including enhanced care, therapeutic decision-making, diagnostic accuracy in the pre-clinical setting, reduced costs, length of stay and patient satisfaction in emergency care (15), and improved treatment adherence, quality of life and physiological variables (5).

Cumulatively, articles invite greater precision in differentiating virtual care design relative to patient and family needs, and targeted benefits or preferences. This supports the careful consideration of virtual care design for specific purposes and populations. For instance, Siani et al. (7) note potential parental preference for “one type of communication technology over the other, for instance, videoconferencing vs. telephone discussion, due to accommodation of visual content, ability to record or repeat messages, and opportunities to ask questions, particularly when parents are not present for direct discussion following physician's daily evaluations” (7) (p.338).

This review, like others, raises questions about the design of virtual care, and the need for greater precision in complementing virtual or hybrid care relative to the specific care needs and ‘fit’ for a given child and family (121, 133). To facilitate this, shared decision-making between children/youth, their families and healthcare providers is necessary to address initial and emergent challenges throughout the design and implementation of virtual care. Study learnings demonstrate that a third of articles addressed design/implementation aspects that affected the accessibility of virtual care (e.g., need for digital equipment, need to speak in a specific language). Consulting with patients and their families (133), and managing families’ expectations of virtual care are highlighted as important in seeking optimal means of offering virtual care (126). This focus on the “fit” of virtual care approaches complements earlier reviews that amplify the need for greater applicability of research to the target population, clinical area and level of acuity (14, 15, 29). In addition, considerations related to “fit” are imperative as virtual care approaches may incorporate additional tasks (e.g., gathering and uploading children's information) that require guidance from the care team/technological staff and time for families to master.

Despite what generally emerged as benefits and preferences for the inclusion of virtual care in contemporary healthcare, caution and substantial research gaps remain. This review as well as other secondary reviews have found insufficient evidence of virtual care utility and outcomes for specific populations; hence, further evidence is needed in addressing outcomes and processes for targeted populations (2, 13, 14, 16)^,^. Like in other reviews (2, 5, 7), heterogeneity and varying rigor were noted in articles. In moving forward, co-design of approaches may contribute to elements of salience for families.

Multiple articles, as reviewed, indicate concerns about SDOH-related inequities in virtual care. As an example, concern was raised about the need to better understand and accommodate logistical issues and reduce costs for patients and families (125, 128, 130) when designing and implementing virtual care, particularly for families in lower income strata (128). Several previous reviews also have identified inequities in virtual care access (14); however, it appears that advances have been made in this area. For instance, Obregon et al. (16) reviewed articles addressing telehealth among families with limited English proficiency. Their review demonstrates the acceptability and feasibility of telehealth in that population; however, health outcomes have not yet been extensively determined. Accordingly, targeted focus is needed with equity, inclusion, and accessibility lenses particularly for children and families from equity-deserving communities as emphasized by Birnie and colleagues (137). Priorities include widespread access to the internet, data plans, and wireless secure Internet hotspots for patients and their families (120, 121), equitable technology access (76), and reimbursement of costs related to virtual care (e.g., insurance payment to healthcare providers and families) (111).

Reviewed articles offer recommendations to improve access, experiences, and benefits of virtual care. In moving forward, robust organizational infrastructure and more support and training for both users and providers of virtual care are invited. This includes staff training, dedicated information technology (IT) support, improved IT applications, facilitators, and increased role clarity (120, 127, 128, 131, 133, 138). In alignment with EDIA, external staff resources such as interpreters and/or culture brokers are critical to help patients, families and/or healthcare providers overcome linguistic and/or cultural barriers that emerge in virtual care. Tailored approaches are required to meet the needs of all children and families (110, 119, 135, 139). Design considerations must accommodate children/families’ levels of comfort with the virtual care approach used, as well as ensuring its accessibility. Facilitating factors identified in this scoping review can be useful in tailoring virtual care approaches relative to needs. Doing so requires the development of a relationship with patients and their families as well as offering support and guidance to families as they transition from in-person to remote care.

Overall, articles included in this review convey over-arching benefits of virtual care, thereby supporting the use of virtual care. But similar to findings of Siani et al. (7), we suggest caution such that virtual care not be viewed as a replacement to face-to-face interaction. Other key takeaways include the urgent need to address SDOH barriers and offer virtual care applications tailored to patient and family needs as well as clinical context. To this end, we emphasize the incorporation of equity frameworks that can better support the design and implementation of virtual care for all.

While current guidelines of the JBI protocol do not incorporate scoping review consultation, scholars such as Arksey and O'Malley (140) and Oravec et al. (141) emphasize that seeking feedback from patients and caregivers for scoping reviews, increases the potential uptake of findings, increased satisfaction and the generalizability of results. We shared our learnings with a broad group of stakeholders, including caregivers, who expressed resonance with the review findings.

### 4.1 Review limitations

Limitations in this scoping review include the following. Search terms did not have variants associated with different online platforms that may have been used in virtual care. Only articles written in English were included, thus omitting potentially relevant information from non-English articles and populations. This gap also may have imposed bias reflective of study selection and language/ethnocultural population exclusion. Examination of sample diversity has not been a focus of this review, inviting future study with a focus on appraising considerations of accessibility in virtual care. In the Canadian context, as per the Truth and Reconciliation Commission of Canada, greater attention should focus on virtual care interventions for Indigenous children/youth and their families.

We acknowledge that this review elicited only articles on patient and family perspectives. We also limited our focus to notions of family satisfaction, preferences and experiences of virtual care. While benefits and challenges were illuminated, studies did not provide demonstrated impacts over time. Ascertaining outcomes (e.g., knowledge uptake, treatment adherence) of virtual and/or hybrid care, and examining the perspectives of healthcare providers and clinical decision makers, warrant further study. Also, the heterogeneity in research designs and methods of virtual care platforms impose complexity in assessing the quality of studies, which was not a focusing this review.

Lastly, the preponderance of articles on virtual care in the last decade is nuanced by the requisite of this shift in the COVID-19 pandemic. Also, the ten years reviewed (2013–2023) have reflected exponential shifts in technology *and* the accessibility of technology. It is recognized that until the pandemic, virtual care options lagged technology industry capacity. Technology advancement (including innovation in artificial intelligence) invites further investigation. Future study also is needed to advance precision in technology capacity and usefulness specific to population, clinical area, and level of acuity.

## 5 Conclusion

Included articles generally convey positive family perceptions about the use of virtual care in pediatrics. However, further research is needed to determine benefits of virtual care for particular populations and under what circumstances those benefits are incurred. Such work is anticipated to offer greater virtual care precision relative to patient and family need. Further refinement of approaches will inform care design; this invites engaging and empowering patients and families to co-design virtual care options. It appears that virtual care indeed is here to stay; thus, it is important to carefully determine care approaches for positive patient and family experience, equitable access and optimal outcomes.
