# Supplementary material for: Synchronous virtual care in children’s health care: a scoping review
Source: Front Pediatr. 2025 Nov 6;13:1610407. doi: 10.3389/fped.2025.1610407 (PMC12631453; doi:10.3389/fped.2025.1610407)
Supplement: Supplementary file 1 [file Table1.docx]

| **Study** | **Country** | **Specialty** | **Virtual Health Term** | **Objective** | **Data Collection** | **Research Design** | **Virtual Care/Telehealth Approach** | **Sample** | **Positive Outcomes** | **Negative Outcomes** |
| --- | --- | --- | --- | --- | --- | --- | --- | --- | --- | --- |
| Akobeng et al, 2015^1^ | United Kingdom | Gastroenterology | Teleconsultation | Aimed to provide evidence about the effectiveness and cost consequences of telephone consultations, compared to face-to-face consultations in childhood IBD | Survey Data | Exploratory | Hybrid (virtual and/or in person) | Caregivers and Children/Youth | Quality of life scores didn't drop, reduced cost and consultation time, reduced wait times at outpatient clinics, safe and cost-effective alternative | Lower consultation time with virtual |
| Alghamdi, 2023^2^ | Saudi Arabia | Pediatric Dentistry | Other | Assess attitudes and responses of parents of pediatric patients towards virtual dental clinics during COVID-19 | Survey Data | Observational (cohort/case study/cross-sectional/prospective/longitudinal) | Multiple/Mixed (ex. video and/or phone calls) | Caregivers | 94% of parents were satisfied with virtual care, felt they could talk to the clinician as well as if they were in-person, save time, easy to use, convenient, risk-free | 3/4 parents had no previous experience with virtual care, technical difficulties for (n=1) |
| Alzahrani et al, 2022^3^ | Saudi Arabia | Endocrinology | Telemedicine | Aimed to assess primary caregiver perceptions of the virtual phone clinics towards routine care of pediatric patients with type 1  diabetes mellitus (T1DM). | Survey Data | Observational (cohort/case study/cross-sectional/prospective/longitudinal) | Telephone Calls | Caregivers | Phone clinics could minimize the probability of COVID exposure and improve diabetes control among patients, majority of patients were satisfied, healthcare cost decreased, enhanced productivity and attendance, facilitated the ability to maintain routine follow-up with physician and enhances accessibility | Only those with internet could complete survey |
| Assenza et al, 2021^4^ | Italy | Rehab (Physical, speech, occupational, cognitive-behavioral, neuropsychological, psychological support) | Telerehabilitation | To explore professionals’, adult patients’, and children’s caregivers’  perception and satisfaction with telerehabilitation during COVID-19 lockdown. | Survey Data | Observational (cohort/case study/cross-sectional/prospective/longitudinal) | Videoconferencing | Caregivers and Children/Youth | Quick activation, maintaining same clinicians, allows parents to maintain daily routines and schedules | Parental anxiety, felt overwhelmed, and worried about child condition was a consequence of telehealth |
| Bales et al, 2023^5^ | United States of America | Pediatric Neurosurgery Clinic | Telemedicine | Determine the feasibility and accessibility of a novel virtual pediatric concussion clinic at a rural academic hospital. | Observation | Observational (cohort/case study/cross-sectional/prospective/longitudinal) | Telephone Calls | Children/Youth | Triaged on the phone allowed patients to have follow-up in their own home, caught serious complications and referred to a specialist they wouldn't necessarily have access to in rural location, saved parents the long drive. | No physical examination to consider during follow-up as it was done on the phone. |
| Bate et al, 2021^6^ | Australia | Multiple specialties | Telehealth | To determine the user and clinician satisfaction during this period and compare to a pre-COVID-19 cohort. | Survey Data | Observational (cohort/case study/cross-sectional/prospective/longitudinal) | Videoconferencing | Caregivers | Good standard of care in TH compared to face-to-face, good level of engagement and standard of care, parents felt safer using TH, major cost savings, saved more time in daily lives, removal of geographical barriers | Some technology barriers |
| Bell et al, 2021^7^ | United Kingdom | Ophthalmology | Telegenetics | To (re-)evaluate families' acceptability of telegenetics. | Survey Data | General Quantitative- unspecified | Telephone Calls | Caregivers | Only 12% indicated that TH did not provide for their needs, received information they needed, appointment was adequate length to address questions, save time, increased access to care, helped those unable to travel | Many patients require a physical examination (especially new patients), anxiety heightened about their child's condition, a majority still preferred in-person consultations |
| Bianciardi Valessina et al, 2016^8^ | Italy | Dermatology | Telemedicine | Can an innovated telemedicine system effectively monitor/care for pediatric patients with complex wounds at risk of infections? | No stated data collection | General Quantitative- unspecified | Videoconferencing | Caregivers and Children/Youth | Satisfaction rate of only 57% in first three months, 84% at the end of the study, Improved treatment options, prevention of wound infections, decrease in stress levels for children, better follow-up rates, Majority of patients experienced wound resolution, Reduced cost, no need to travel for those far from hospital | Parents were hesitant to adopt TH |
| Blagdon et al., 2022^9^ | Canada | NICU | Virtual Communications Initiative | To evaluate the overall experience for families and staff with virtual communications in a Level III NICU in Calgary. | Survey containing structured and free text responses | Mixed Methods | Video Calls (Zoom) via tablet | Parents | - Facilitators: Informational handouts and a short video with FAQs were given to families.  - Relationship between parent and child: Parents strongly agreed that participation in the intervention had a positive impact on them and their infant.  - Satisfaction: 100% of parents said that it met their expectations and 90% of parents said it was an 'excellent' or 'good' experience. | - Decision-making: Some did not feel like they were able to make decisions about their child’s care plans  - Presence: Difficult not to touch child via video.  - Technical: Video was okay, but audio was poor. |
| Brewster et al, 2023^10^ | USA | Multiple specialties | Telemedicine | Describe the operationalization of the program and evaluate the effect of internet and tablet use on virtual missed care opportunities, virtual visit modality, and perceived care experience. | Survey Data | Exploratory | Multiple/Mixed (ex. video and/or phone calls) | Caregivers and Children/Youth | Improvements of care, High degree of satisfaction from parents, decrease in missed care opportunities, ease of use | Yielded a cohort most affected by the 'digital divide' - racial and ethnic minoritized group, non-English speaking, and/or publicly insured, 28% of families required tech support, bad internet connection, damage to tech |
| Brothwood et al., 2021^11^ | United Kingdom | Psychiatry (Eating Disorders) | No term used | To understand the experience of being involved in an intensive treatment program online during the COVID-19 pandemic from the perspective of young people and their parents | Survey containing structured and free text responses | Mixed Methods | In-person and online | Adolescents and their parents | - Benefits: Both parents and adolescents felt online option offered opportunity to receive care during the COVID-19 pandemic;  - Satisfaction: Parents and adolescents felt intervention could be accessible anywhere. However, parents were more comfortable, and had more positive experiences compared to adolescents; Some adolescents felt online sessions were productive and easy to engage with  - Preferences: Youth wished to return to in-person meetings, but parents did not mind or preferred online support. | - Quality of care: Compared to in-person formats, online was not necessarily the best option; some adolescents did not enjoy online components.  - Preferences: Youth wished to return to in-person meetings  - Presence: Parents and adolescents had relational challenges with platform (e.g., screen is impersonal, lack of physical touch, difficulty to process emotions, needing to learn new ways to interact)  - Technical: Adolescents experienced technical difficulties |
| Bullock et al, 2017^12^ | United States of America | Rheumatology (Inflammatory) | Telemedicine | The general objective is to explore the barriers of pediatric rheumatology care and to explore the acceptability of alternative pediatric rheumatology care models like telemedicine. | Survey Data | Exploratory | No information given | Caregivers | Decreased travel cost | 95% reported preference for in-person vs. virtual |
| Carretier et al., 2023^13^ | France | Psychiatry | Telehealth | To explore the experience of telehealth during the COVID-19 pandemic | Interviews | Qualitative (Phenomenology) | Hybrid (Online and In-person) | Adolescents and Parents | - Benefits related of receiving telemedicine: Choice in what platform to participate in; Parents became more involved  - Caregiver Outcome: Helped parents be more involved in their child’s care  - Effectiveness: Teleconsultation was acceptable for those who were stable | - Effectiveness: Telemedicine did not work for those who had major instability  - Ethics: Concerns about privacy and confidentiality  - Re-learning: Loss of contextual elements resulted in adolescents learning new behaviors (e.g., how to be safe, knowing when to have a consultation) |
| Caruso et al., 2023^14^ | United States of America | Neonatal Intensive Care Unit | Remote patient monitoring (another form of telemedicine) | To examine parents' experiences of using ‘Growing at Home’: a remote patient monitoring program for stable infants who were discharged from the NICU. | Individual Interviews | Qualitative | Tablet and telephone | Mothers and infants | - Benefit: The remote intervention enabled mothers to feel relaxed, comfortable and do desired activities. The remote intervention also enabled mothers to return home early, to save time and costs on transportation and accommodations.  - Child’s health: Mothers felt their infant was doing well.  - Satisfaction: Mothers felt there was a learning curve but felt supported by team. They felt the technology was easy to use and gathering their child’s data was easy. All felt fully prepared for the discharge at home and comfortable with the protocols for the intervention. | - Engagement: Some families experienced challenges due to mixed messages given by their child’s providers or when responsibilities were not well-defined.  - Preferences: Many parents felt the program did not fit the needs of every family or infant and that families need to be well prepared and comfortable before taking part in the intervention.  - Satisfaction: Two families felt it was difficult to manage child concerns over the phone such as managing reflux or vomit. Some felt they weren’t well prepared to do required outpatient appointments. There were mixed thoughts about the phone where some felt comfortable about the phone, and some felt it was unnecessary. |
| Castro et al., 2022^15^ | United States of America | Mental Health (Child and adolescent mental health) | Telehealth | To explore the experience of transition to remote care | Focus Group Interviews | Qualitative | No information given | Adolescents and Parents | - Not applicable. | - Ethics: Youth had privacy and safety concerns  - Technology Literacy: Parents had difficulty using technology due to limited literacy and lack of growing up in a technology culture |
| Chalmers et al, 2018^16^ | Australia | Oncology | Telehealth | Assess the feasibility and acceptability for both patients and clinicians of providing a psychosocial assessment via telehealth to adolescents and young adults currently receiving treatment for cancer, relative to face-to-face delivery. | Survey Data | Experimental | Videoconferencing | Children/Youth | Reduced burden of travel, increased ease of attending assessment | Language barrier (n=1) |
| Chan et al, 2022^17^ | Hong Kong | Nephrology (kidney care) | Other | To provide prospective data on important clinical outcome measures, such as hospitalization rates and blood pressure control, and evaluate patient quality of life among children receiving automated peritoneal dialysis (APD) with remote patient monitoring (RPM). | Observation | Observational (cohort/case study/cross-sectional/prospective/longitudinal) | Multiple/Mixed (ex. video and/or phone calls) | Children/Youth | Fewer unplanned hospitalizations, shorter hospital stays, trend of reduction in diastolic blood pressure, sense of security, better quality of life | Not applicable. |
| Charnell et al., 2020^18^ | United Kingdom | Surgery | Remote Consultation | To explore the experience of a remote clinic during the COVID-19 pandemic | Focus Group Interviews | Qualitative (Service Evaluation) | Video and Telephone Consultations | Families | - Satisfaction: Parents were positive about their experience.  - Preference: Parents preferred remote consultations were beneficial for the future. However, there were mixed perceptions about the platform. Those who had a phone consultation felt video consultation would not have enhanced their appointment; Those who had a video consultation preferred a video consultation as they never met the provider | - Engagement: Some parents had difficulty retaining information and some felt having a pen and paper would have been beneficial  - Ethics: Parents were concerned about what information provider wanted |
| Cockrell et al, 2022^19^ | United States | Surgical Care | Telehealth | Examine rates of utilization and satisfaction with telehealth among Hispanic patients. | Survey Data | Observational (cohort/case study/cross-sectional/prospective/longitudinal) | Videoconferencing | Caregivers | Not applicable | Spanish speakers had less time spent with providers compared to English speakers, Lower satisfaction with providers explanation of the patient's health, listening to families concerns, and time spent on TH, Low response rate, virtual interpreter service was difficult to use |
| Costa-Cordella et al, 2023^20^ | Chile | Mental Health (Child and adolescent mental health) | Zoom | To investigate the effectiveness of a digital adaptation of an outpatient care service for children and adolescents with mental health issues | Semi-structured interviews and questionnaire | Mixed Methods | Zoom | Children/Youth | -Benefits: Young participants had social benefits (e.g., exchange stories and share emotions, meet new people), psychosocial benefits (e.g., more sociable, happier, less worried, felt safe and respected), logistical benefits (e.g., not needing to travel),  - Satisfaction: Young participants were 100% satisfied with mental health intervention with half stating they were very satisfied and the remainder stating they were satisfied | - Privacy: A few participants felt that there was not enough privacy.  -Technology: One young participant stated there was a problem connecting to the internet and a few could not connect as they did not have computers in the home |
| Darr et al, 2020^21^ | United Kingdom | Otolaryngologist (Ear, nose, and throat) | Telemedicine | To evaluate the impact of the COVID-19 pandemic on paediatric otolaryngology outpatient services whilst collating patient feedback to elicit long-term sustainability post COVID-19. | Survey Data | Observational (cohort/case study/cross-sectional/prospective/longitudinal) | Multiple/Mixed (ex. video and/or phone calls) | Caregivers and Children/Youth | Minimizing viral exposure, appointments not cancelled due to covid, Subtle reduction in discharge rates indicative of examination limitations, no show rate decreased, preferable for patients who had to travel further | Privacy issues need to be addressed, relevant tech needs to be had by patients |
| Davis et al, 2021^22^ | United States of America | Cardiothoracic | Telehealth | aimed to achieve a virtual visit volume of at least 75% of our pre-pandemic volume. We also describe patient and provider experience with telehealth services. | Survey Data | General Quantitative- unspecified | Multiple/Mixed (ex. video and/or phone calls) | Caregivers and Children/Youth | Highly satisfied and convenient, Patients able to see all providers needed, had adequate time, and felt questions were answered, | Moderate concern about the lack of physical examination and testing, low response rate. |
| Dempsey et al., 2021^23^ | United States of America | Gastroenterology | Telehealth | To define factors influencing family's perceptions of telehealth and secure feedback on the telehealth experience | Survey containing structured questions (Likert scale) | Mixed Methods | Telephone | Children and/or their parents | - Benefits: Convenience, did not have to miss time away from work, decreased costs related to in-person visits, early appointment, continuity of care despite experiencing technical issues  - Satisfaction: 97.5% felt telehealth was beneficial; acceptance of telehealth was largely contingent on circumstances emerging from the COVID-19 pandemic (e.g., only option of care); those who had a higher income bracket did not disagree or were not neutral about their telehealth experience | - Financial and Time Costs: Resources (e.g., weight scale) needed to be purchased by parents; home and family demands (e.g., home distractions)  - Quality of Care: Difficulty to do proper assessments due to lack of in-person consultation; accuracy of objective data collected at home  - Satisfaction: 46.25% were neutral or disagreed that their telehealth experience was good if they received an office visit |
| Doerdelmann, 2022^24^ | Germany | Endocrinology | Video Consultation | To investigate the experience of adding monthly and supplementary video consultations on standard care that is delivered over one year | Phone interviews | Qualitative (Evaluation) | Video (Used to upload information) | Families | - Benefits: Parents felt video format was relaxing, safe from contracting COVID-19, and could connect with care team via email; Parents felt they could save time.  - Child’s health: Ongoing care (e.g., high-frequency counseling and closer monitoring) benefited child’s glucose trends  - Caregiver Outcomes: Improved parent’s knowledge of child’s diabetes and emotions (e.g., a sense of calm) related to being a caregiver  - Preference: Parents felt they could fit a video consultation in their daily lives.  - Satisfaction: Parents felt telemedical care was the same as outpatient contact; Parents appreciated flexibility in doing a virtual consultation; Parents were satisfied with care | - Quality of care: Lack of a physical examination  - Technical: 2/3 of parents had difficulty connecting with internet |
| Edwards & Parry, 2022^25^ | Ireland | Outpatient | Telemedicine | To evaluate the acceptability of telephone consultations as an alternative to conventional paediatric outpatient appointments  and assess whether it could continue to have a useful role beyond the pandemic | Survey comprising demographic information, structured responses (Likert scales, yes/no scales) and free texts | Mixed Methods | Telephone | Caregivers | - Satisfaction: Among parents, 97.6% felt safe; 93% felt their expectations were met during the consultation; 68.1% felt telephone consultation was the same as an in-person consultation; 86.4% had no problems with the telehealth consultation; Parents satisfied with clinician’s behavior (e.g., professional tone and doing a careful history taking)  - Preferences: Preference for telehealth is based on patient care needs (e.g., child does not have acute needs), patient age (e.g., suitable for older children), parent expectations (e.g., when parents are not expecting their child to have a clinical examination), family home region (e.g., if family has to travel longer distances) | - Administrative: Receiving incorrect contact information, not obtaining appropriate pre-appointment letters, calling at the wrong time  - Presence: Lack of assessment; lack of in-person interactions  - Technical: Connectivity and audio issues |
| Elbin et al, 2022^26^ | United States of America | Rehab (Physical, speech, occupational, cognitive-behavioural, neuropsychological, psychological support) | Telehealth | Pilot study of caregiver ratings of therapeutic alliance and patient satisfaction outcomes  between telehealth and in-person concussion clinical care in male and female adolescent athletes | Survey Data | Experimental | Videoconferencing | Caregivers and Children/Youth | High satisfaction rating, Concussion clinical evaluation are possible via TH, patient comfort. | Only included one clinical visit |
| Finkelstein et al, 2020^27^ | United States of America | Urology | Telemedicine | We compared virtual visits, i.e. remote patient encounters, via a live video system, with conventional in-person visits with respect to clinical outcomes,  family experience and costs in a pediatric urology surgical population. | Survey Data | Observational (cohort/case study/cross-sectional/prospective/longitudinal) | Videoconferencing | Caregivers | High satisfaction rating, Waiting room time was cut in half, more time with physician, time saver (15 minutes instead of 250 minutes), more convenient, no cost compared to in person, missed less school/work, Children were able to spend more time in school fostering relationships with peers and more time with their families at home, No TH patient required an in-person visit for further evaluation | Appointment compliance rates weren't as high as in-person visits |
| Finnegan et al., 2022^28^ | Ireland | Outpatient | Telehealth | To explore the experiences of families of children attending a neurodevelopmental service after a transition to virtual  consultations | Survey | Mixed Methods | Telephone | Caregivers | - Satisfaction: Parents were satisfied with face-to-face and virtual consultations; many parents were satisfied with their engagement in the virtual consultation (e.g., 97% felt they had adequate time; 86% felt their concerns were managed and received adequate/correct information)  - Preferences: 76% of parents were happy with a hybrid (e.g., virtual and in-person) appointment in the future | Not applicable |
| Fletcher et al., 2021^29^ | Canada | Paediatrics (General) | Not Applicable | To gather the perspectives from youth regarding virtual appointments during the COVID-19 pandemic | Focus Group Discussions | Qualitative | No intervention | Children/youth | - Benefits: Youth felt virtual consultations enabled continuity of care during COVID-19 pandemic and created safety from contracting COVID-19; Youth felt virtual platforms were accessibility and saved time.  - Preference: Youth preferred virtual consultation particularly from youth who faced specific barriers (e.g., live in far regions); Preferences for virtual consultation were contingent on nature of appointment and delivery of care, and inclusion of youth perspectives in implementing a consultation. | - Ethics: Youth did not have a safe space and could not describe their symptoms accurately  - Engagement: Youth lost ‘embodied’ connections with providers and peers due to lack of in-person appointments  - Technology accessibility: May be difficult to implement if youth do not have reliable access to technology and internet. |
| Fortini et al, 2020^30^ | Argentina | Neurology | Telemedicine | This study's aim was to assess parent satisfaction three years after implementing a pediatric epilepsy telemedicine program. | Survey Data | Observational (cohort/case study/cross-sectional/prospective/longitudinal) | Videoconferencing | Caregivers | All families satisfied with the program, Seizure frequency decreased more than 50% in 96% of the patients, 10 patients had become seizure free, better access to medication, Majority felt they had received clear information about the epilepsy of their child, More regular follow-up with shorter intervals between visits, saved substantial travel costs and travel time, less work and school time lost, Families felt supported by the team, Valuable asset for patients living in remote rural areas and resource poor countries | Some felt embarrassed in front of the camera, lack of privacy |
| Foster et al, 2020^31^ | United States of America | Emergency Medicine | Telemedicine | When a family was involved in the consultation, parents (guardians) were surveyed by phone within 72 hours, to understand their experience. | Survey Data | General Quantitative- unspecified | Multiple/Mixed (ex. video and/or phone calls) | Caregivers | 86% indicated that TH was helpful, 85% felt confident in care received, can improve access to care, family doesn’t have to travel away from home. | Tech issues, more staff training, different clinical management, improved language interpretation |
| Frye et al, 2022^32^ | USA | Psychology | Telehealth | What are the differences in access to care for patients using MTH including travel, cost savings, and show rate? | Survey Data | Observational (cohort/case study/cross-sectional/prospective/longitudinal) | Hybrid (virtual and/or in person) | Caregivers and Children/Youth | Saved cost, don't have to drive far distances, increased access to care, patients showed for more virtual sessions than in-person sessions | Internet quality or platform connectivity did not allow some to join sessions |
| Gan et al., 2021^33^ | United States of America | Urology | Telehealth | To explore the expansion of a telemedicine intervention for pediatric urology visits | Surveys had structured and free-text questions | Mixed Methods | Web application accessed via tablet or telephone | Family but referred to as children and their caregivers | - Benefits: Save on travel, do not miss work or school; continuity of care  - Satisfaction: Many families were satisfied with video visits and perceived visits met their child’s medical needs  - Preference: 90% of families felt they would strongly recommend a telehealth visit to other families | - Satisfaction: 10% had mixed perceptions that video visits were convenient but felt a need to do an in-person visit.  - Technical: 15.6% families experienced audio-visual issues; 7.6% had internet connectivity issues |
| Garne Holm, 2019^34^ | Denmark | NICU | Telehealth | To explore the experience of doing a tele-homecare service | Focus Group Interviews | Qualitative | Video and Tablet (Used to upload information) | Parents | - Benefits: Parents did not have to worry about their appearance; Parents did not have to travel  - Caregiver outcomes: Parents felt virtual platform helped them feel better as a caregiver (e.g., improved ability to make decisions) and gave them sufficient information to monitor their child’s progress  - Relationship between parent and provider: Parents felt listened to and validated by provider, had a provider who demonstrated professionalism and expertise  - Relationship between parent and child: Parents felt closer to child, practice equal dynamics between spouses, address other care-giving issues  - Satisfaction: Parents felt satisfied, safe, calm, a sense of closeness and it was natural to talk via a virtual platform | - Not mentioned |
| Gefen et al., 2021^35^ | Israel | Rehabilitation (Includes physical therapy, occupational therapy, speech therapy and psychology) | Telehealth | To explore the advantages and disadvantages of receiving a telerehabilitation treatment | Surveys + Medical data + Focus groups | Mixed Methods | Video Calls (Zoom) or Telephone | Children and their caregivers | - Benefits: Flexibility in making appointments, convenience (e.g., save on travel time)  - Relationship between parent and child: Caregiver engaged in child’s care (e.g., can discuss progress of child, feeling motivated to learn); Having more alone time with child; Can collaborate with child to implement goals  - Relationship between parent and provider: Collaborate with clinician on treatment goals | - Ethical Issues: Lack of privacy  - Quality of Care: Caregiver not agreeing with clinician on aspects of treatment (e.g., treatment goals and its implementation)  - Technology: Fears of technology |
| Giuseppe et al, 2022^36^ | Italy | NICU | Telemedicine | Aimed to evaluate satisfaction and stress levels between parents in the telematic family-centered care group versus the FCC group and the no Family-Centered Care group. | Survey Data | Experimental | Videoconferencing | Caregivers | Good satisfaction level, Parents felt more included in healthcare team, Parents opinions about the baby are heard from the entire team, video call with baby gave emotional connection b/w parents and child | Sight of a needle or tube put in their baby surrounded by machines had a negative impact on the groups. |
| Gund et al., 2013^37^ | Sweden | NICU | eHealth | To explore the use of eHealth to improve the communication between parents of premature infants and home health care nurses and to learn how eHealth enhance families' confidence in their care of their child | Surveys (Likert scales and yes/no scales) + Interviews | Mixed Methods | (1) In-person, (2) In-person + support accessed through webpage and (3) In-person + support accessed through Skype | Mothers | For those receiving home care + virtual support via webpage:  - Benefits: Ease in connection; webpages offer updated information, visualization of child’s progress and reminders of appointments; caregiver felt more confident in caring for child; Reduction in home visits; Nurse provided explanation on home visits when filling out data  - Relationship between parent and provider: Parents felt providers were very competent, friendly, and they had time to ask and discuss questions/issues  - Preferences: 83% felt web application was easy to use and felt the application was good or very good   For those receiving home care + virtual support via Skype:  - Benefits: Visualization improved communication and increased confidence in caring for child; reduction in home visits with some stating that video calls could replace home visits  - Satisfaction: All families felt Skype was easy to use, most (75%) felt conversations with the provider was easy and they received answers to questions; Half felt Skype was less stressful than home visits | - Preferences: A third of families wanted to have additional features on the webpage namely in the areas of communication (e.g., direct contact with provider via chat), management (e.g., providing email reminders to provider to respond to questions) and resources (e.g., offering additional links to sites to increase parent’s knowledge of child’s condition and trajectory) |
| Guttmann-Bauman et al, 2018^38^ | USA | Endocrinology | Telehealth | Improved access to care through telehealth clinics would 1) increase the number of yearly visits to our clinic, 2) decrease visits to emergency departments and hospitals, and 3) improve metabolic control and compliance with recommended screening procedures. In addition, they believed that 4) telehealth visits would not be inferior to face-to-face care from the patient/family perspective. | Survey Data | Exploratory | Videoconferencing | Caregivers and Children/Youth | High satisfaction rating, have not seen an increase in metabolic control, Significant decrease in missed work and school hours. Improved access to services. | Not yet have the capability to include the entire team in the consultation, hard to obtain optimal control in the teenage population |
| Hale et al., 2023^39^ | United States of America | Critical Care and Pain Medicine | Virtual Format | To explore the feasibility and acceptability of a virtual adaptation of a workshop for chronic pediatric patients | Surveys and user data | Mixed Methods | Unknown | Adolescents and caregivers | -Benefits: 83.1% of caregivers compared to 72% of adolescents were more likely to be optimistic about the potential effectiveness of the intervention.  -Preferences: Of adolescents, 71.9% were willing to use a virtual platform and 91.5% felt they were supported, 87.9 % felt it was easy to participate and 79.7% felt they could support others. For caregivers, 93.3% were willing to use a virtual approach and 79.4% felt virtual sessions were better than in-person sessions. Also, 93.4% felt supported during the intervention and 80.7% felt they could support others.  - Satisfaction: Most adolescents and caregivers felt intervention addressed concerns with chronic pain. Adolescents had confidence about the learned material and 93.1% were successful in making their own care plan. 91.4% of adolescents were satisfied for completing the intervention, 91.5% enjoyed the intervention, 79.3% were confident in managing their pain following the intervention and 94.8% planned to make changes in their daily routine.  - Technology: 81% of adolescents and caregivers had no technology issues | -Preferences: 62.3% of adolescents preferred in-person compared to virtual sessions.  - Technology: Only one family experienced and had to go to the hospital to access the intervention. 16% of families had video connectivity issues and 14.4% experienced audio lags. |
| Hallford et al, 2022^40^ | USA | NICU | Telemedicine | Current study assessed both health care providers' knowledge of and satisfaction experienced by those families who participated in this service. | Mixed data collection (interviews + observation) | Exploratory | Hybrid (virtual and/or in person) | Caregivers | Good satisfaction level, Ability to be 'present' for their child's care | Privacy and confidentiality with TH system as NICU is an open bay unit |
| Heath et al, 2015^41^ | USA | General Pediatric | Other | To see if telemedicine can effectively monitor care | Survey Data | General Quantitative- unspecified | Telephone Calls | Caregivers and Children/Youth | Help in stressful transition to home care. Follow-ups necessitated in a significant intervention. Reduction in readmissions. Increased access to care, Post discharge phone calls can resolve many important issues | Patients unable to be reached may be the group that is most disconnected and at highest risk for adverse outcomes |
| Hendra et al, 2021^42^ | USA | Cardiothoracic | Telemedicine | To explore satisfaction of telemedicine | Survey Data | Observational (cohort/case study/cross-sectional/prospective/longitudinal) | Hybrid (virtual and/or in person) | Caregivers | 80% satisfied with TH visits, avoiding exposure to covid, providers have more in depth and focused conversations with caregivers, Clinic no show rate improved by 40%, Decreased travel time and cost, no need to arrange for childcare | Difficulty obtaining throat cultures, lab testing, and mental health screening. |
| Hiscock et al, 2022^43^ | Australia | Multiple specialties | Telehealth | To explore satisfaction of telemedicine | Survey Data | Observational (cohort/case study/cross-sectional/prospective/longitudinal) | Hybrid (virtual and/or in person) | Caregivers | Improves access to care, no risk of sickness caught in hospital. Cost savings, saved, time no travel, convenient, no time off work for parents and school for kids | Children in developmental, surgical, or behavioural concerns did not agree that TH was as good as in person. Tech issues, may increase costs from a gov't perspective |
| Hoi et al, 2022^44^ | USA | Otolaryngologist (Ear, nose, and throat) | Telemedicine | Telemedicine visits would have a higher cancellation rate and shorter cycle time than in-person visits. | Survey Data | Observational (cohort/case study/cross-sectional/prospective/longitudinal) | Multiple/Mixed (ex. video and/or phone calls) | Caregivers and Children/Youth | 100% positive reviews from all TM surveys, more appointments with shorter cycle times, less cancellations for TM visits, High satisfaction with care 100% for virtual appointments, Saved travel, | Higher no-show rates, some said that TM limited patient care due to no physical exam |
| Holzman et al, 2021^45^ | USA | Urology | Telemedicine | Reduced waiting time and convenience associated with telemedicine visits provide an opportunity for telemedicine as a useful modality for pediatric urology. | Survey Data | Observational (cohort/case study/cross-sectional/prospective/longitudinal) | Videoconferencing | Caregivers and Children/Youth | 92% satisfaction virtual. 87% in person visits. More face time with physician, increase visits/year while taking away risk, less stressful, less school missed, helps financially on parents, reduced wait time, convenience, Higher satisfaction rate than in-person appointments | Adoption with new technology, low-income families have more difficulty downloading and utilizing software |
| Hoyt-Austin et al., 2022^46^ | United States of America | NICU | Videoconferencing | To explore the experience of lactating parents of hospitalized preterm infants using video-conferencing technology to connect with infant and how that affected their milk pumping experience | Individual Interviews | Qualitative | Video | Children and Parents | Positive  - Benefits: Video enhanced families’ understanding of the complexity of the care for child  - Caregiver outcome: Moms felt productive as a caregiver  - Relationship between child and parent: Increased bonding and connection between parent and child  - Satisfaction: Mom felt relaxed, lower level of stress, comfort and enjoyment | Negative  - Engagement: One felt parent-baby bonding via video may be difficult for young infant compared to older infant  - Psychosocial outcome: Video illuminated to moms the severity of the child’s condition, the difficulty of not present for the infant and the challenge of not knowing when child will be discharged. |
| Huscsava et al., 2022^47^ | Austria | Psychiatry | Teletherapy | To explore ths success of a teletherapy delivered to adolescents during quarantine instead of receiving the therapy in-person | Surveys containing closed-ended and open-ended questions | Mixed Methods | Video calls (Insta-help) | Adolescents | - Satisfaction: In a crisis, only 37% felt teletherapy was better than in-person encounters. 36% felt neutral | - Functioning: Those who had a deterioration in their psychosocial functioning felt teletherapy was unsafe, superficial and less private. Further, those who had higher amounts of rumination and introspection reported more concerns with their emotions and the response of the crisis team.  - Satisfaction: During a crisis, 27% felt teletherapy was worse on their personal well-being and symptom improvement.  - Technical: All participants reported technical issues. |
| Hylen et al., 2022^48^ | Sweden | NICU & Surgery | eHealth | To explore the experience of supplementing care with eHEALTH | Interviews | Qualitative | Hybrid (Video, Telephone, Chats and a Tablet to upload information) | Children and Parents | - Benefits of using a tablet: Parents felt tablet offered ongoing communications with child’s care team online and offline, documented child’s progress, easy for parents to upload information, ensure safety during COVID-19 pandemic, cut down on travel  - Caregiver outcome: Parents felt validated as a caregiver by provider  - Technological Literacy: Parents’ acceptance of tablet attributed to growing up in a generation that approves tablets. | - Challenges using a phone: Parents felt the phone was difficult as they could not get in touch with the right person, did not want to infringe on providers’ time, saw calls as time-consuming and difficult  - Challenges using a tablet: Parents felt tablet was challenging due to wait times for responses, technical issues (low battery), uncertainty in uploading private information without a clear digital roadmap and could not provide accurate pictures of child’s condition |
| Jones et al, 2022^49^ | USA | General Pediatric | Telemedicine | Parents/caregivers would be less interested in using telemedicine again if their children were younger, were seen for "hands on" therapy services, and/or lived near the hospital. | Survey Data | Observational (cohort/case study/cross-sectional/prospective/longitudinal) | Multiple/Mixed (ex. video and/or phone calls) | Caregivers and Children/Youth | High levels of satisfaction in initial months and after it's onset, Less stress, less travel | Selection bias due to low and inconsistent response rate across timepoints and sampling, Distractions at home, hard to get child to pay attention |
| Joseph et al., 2022^50^ | India | Surgery | Telemedicine | To explore the experience of accessing support via email/telephone/what's app during the COVID-19 pandemic | Not known | Qualitative | WhatsApp and Hospital App (View hospitals and book appointments) | Children and Caregivers | - Benefits: Parents felt telemedicine enabled communication with providers, send health reports to nearby health centers, enable visual examinations by provider and created a sense of safety during COVID-19 pandemic  - Child’s health: Parents felt telemedicine facilitated child’s physical health  - Relationship between provider and parent: Parents felt heard and listened to by providers.  - Satisfaction: Parents felt telehealth were positive due to visual contact and ability to observe body language | - Marketing of intervention: Parents felt responsible for increasing awareness of telemedicine.  - Technical: Parents experienced delays, connectivity, or network issues |
| Jury et al, 2013^51^ | Australia | NICU | Telehealth | Is telehealth suitable for both simple and highly complex pediatric patients? | Survey Data | Observational (cohort/case study/cross-sectional/prospective/longitudinal) | Videoconferencing | Caregivers and Children/Youth | Diagnosis of simple tics, investigation of allergy, and follow-up, chance to see specialists, Save family time, innovative | When clinician is not convinced of clinical safety or benefit, technical challenges |
| Kelly et al., 2019^52^ | United States of America | Transplant | Telehealth | To improve access to care and engage adolescent transplant recipients for adherence-promoting interventions, innovative solutions utilizing technology as needed. | Surveys containing closed-ended and open-ended questions | Mixed Methods | Desktop camera and video-conferencing software | Adolescents | - Benefits: Reduction in travel (83%) and missing school/work (67%); Create opportunities to connect with others  - Child’s outcome: Telehealth helped participants manage medication use and create coping strategies for health | - Technical: Internet connectivity issues, audio challenges  - Preferences: Need to tailor meetings (e.g. allow open-ended responses, have an in-person meeting at the end of treatment, have a tablet/laptop to see everyone) |
| Khoury et al, 2020^53^ | Canada | Cardiothoracic | Telemedicine | Can a high-interval training protocol using the MedBIKE in a pediatric Fontan population assist in their overall health and is it feasible, safe, and enjoyable? | Observation | Observational (cohort/case study/cross-sectional/prospective/longitudinal) | Videoconferencing | Caregivers and Children/Youth | Height and weight recorded along with oxygen consumption values, easy to navigate, Less travel | Uncomfortable mouthpiece and headgear on throughout the assessment, 1 patient was depressed, tech connection problems |
| Kilipiris et al, 2023^54^ | United Kingdom | Craniofacial Surgery | Telemedicine | Evaluate the satisfaction level of parents from telemedicine use in the long-term follow-up of children operated for craniosynostosis during the COVID-19 pandemic. | Survey Data | Observational (cohort/case study/cross-sectional/prospective/longitudinal) | Videoconferencing | Caregivers | Parents were satisfied with the program, children were able to be diagnosed from home, parents felt that the physician's advice was helpful, convenient to use. | Only 18.7% would prefer telemedicine consultations in the future, no physical examination for their child, technical difficulties |
| Kolb et al, 2021^55^ | United States | Otolaryngologist (Ear, nose, and throat) | Telehealth | Determine the rates and primary causes of missed appointments for telehealth visits and present remedies for improvement | Survey Data | Observational (cohort/case study/cross-sectional/prospective/longitudinal) | No information given | Caregivers and Children/Youth | Not applicable | TH patients far more likely to miss an appointment compared to in-person, Sig difference in race/ethnicity and insurance categories of missed appointments, tech issues accounted for 50% of missed appointments |
| Kramer & De Asis, 2021^56^ | United States | Osteopathy | Telehealth | Demonstrate experience with osteopathic interventions via telehealth and perhaps create an avenue for patient treatment when distance, illness, or other factors prohibit an in-person OMT visit | Survey Data | Observational (cohort/case study/cross-sectional/prospective/longitudinal) | Videoconferencing | Children/Youth | Physician could demonstrate technique over video so families could visualize, no significant difference in pain reduction for in person or TH visits, Safety and flexibility, avoid viral exposure, no serious complications reported | Some felt uncomfortable performing osteopathic interventions, Hard to instruct parents of TH platform |
| Lakshin et al, 2021^57^ | Germany | Surgical Care | Telemedicine | explore the practice of telemedicine in pediatric surgery in Germany, the impact of the pandemic on its development and parents' and surgeons' experiences with telemedicine | Survey Data | General Quantitative- unspecified | Multiple/Mixed (ex. video and/or phone calls) | Caregivers and Children/Youth | Overall positive experience, 96% trusted physician, Saved time and resources | Lack of physical exam, hard for the child to participate due to anxiety of not knowing the doctor |
| Lanzarin et al., 2021^58^ | Brazil | Outpatient | Telemedicine or Teleconsultation | To explore the experience of teleconsultations for pediatric follow-ups during the COVID-19 pandemic | Individual Interviews | Qualitative | Website portal (Do consultations via portal) | Parents | - Satisfaction: Parents enjoyed the teleconsultation | - Technical: Login challenges when accessing chats; not knowing how to follow guidelines to enter chats; not being able to use chat; not having access to internet |
| Lawrence et al, 2022^59^ | Australia | Multiple specialties | Teleconsultation | 1. to understand parental awareness and appetite for virtual health modalities, including asynchronous communication and remote monitoring.  2. to understand which socio-demographic characteristics are associated with greater awareness and appetite for virtual health | Survey Data | General Quantitative- unspecified | Multiple/Mixed (ex. video and/or phone calls) | Caregivers | Parents more likely to consider asynchronous communication if they reported prior experience of teleconsultation | Terminology used to assess awareness of virtual health care may have been difficult for some parents to interpret, older parents had less appetite for synchronous healthcare, language barriers |
| Lee et al., 2023^60^ | Canada | Chronic Pain | No term used | To examine the outcomes of a cognitive-behavior therapy-based online versus in-person delivered to parents | Structured surveys | Mixed Methods | Does not provide details on format | Parents | - Benefits: Create opportunities to connect with others  - Caregiver outcome: Being part of the virtual support group provided improvements in flexibility, protectiveness, distress and self-regulation  - Satisfaction: Parents were satisfied with both the virtual and in-person support group but there were no differences. | - Preferences: 14% did not prefer the virtual format |
| Lindkvist et al., 2021^61^ | Sweden | Neonatal and Surgery | eHealth | To describe parents' experiences and usage of an eHealth solution as their child transitions from hospital to home following surgery or preterm birth | Interviews and Usage data | Mixed Methods | Intervention delivered through a tablet and offered video, texting and repository for parent to upload child's data | Mothers | - Benefits: Device has numerous benefits such as easy access, addresses non-acute concerns, offer instant feedback, continuity of care, safety, follow child’s progress  - Caregiver outcome: Device was essential in supporting how they care for their child and coping with worry.  - Relationship between parent and provider: Parents felt less stressful in communicating with nurse | - Lost Data: If pictures were deleted, some felt parents could not make reference to them during a consultation.  - Preferences: Need upgrades like physical security (e.g., have a protective cover), useability (e.g., improved touch sensitivity, change interface) and additional programs (e.g., link child’s medical journal to device)  - Responsiveness of Support: On the device, parents were uncertain when their questions would be answered.  - Technical: Battery not reliable, lost connection, login issues, varying quality of video  - Technical Support: Some parents felt technological support from nurses was not timely or lacking. |
| Lo et al, 2021^62^ | United Kingdom | Surgical Care | Teleconsultation | Evaluate effectiveness of TC and parents' experience with neurosurgeon- and CNS-led TC compared with the traditional FTF clinic encounters and identify factors, if any, associated with their preferences | Survey Data | Observational (cohort/case study/cross-sectional/prospective/longitudinal) | Telephone Calls | Caregivers and Children/Youth | 64% families preferred the next appointment to be on the telephone, Overall appt was same/better/much better using TH | Not applicable |
| López Seguí et al, 2020^63^ | Spain | Multiple specialties | Other | Assess families' degree of satisfaction and acceptability of pediatric tele-homecare and explore the clinical characteristics of children benefiting from the program | Survey Data | Experimental | Telephone Calls | Caregivers and Children/Youth | Did not generate significant adverse incidents, 100% would repeat the experience, level of care was seen as Excellent (92%), allowed families to be self-sufficient, improvement on quality of life of the child and the family, software was perceived as easy (81%) | Challenge with device size. |
| Love et al, 2022^64^ | United States | Gastroenterology | Telemedicine | 1. to assess patient and caregiver perceptions about utilizing the telemedicine platform for the outpatient clinic visit  2. to describe differences in clinical management provided via telemedicine as compared to in-person clinic | Survey Data | General Quantitative- unspecified | Hybrid (virtual and/or in person) | Caregivers | 89% planning to use TM again, only 19% preferred an in-person appointment due to covid, increased access to care, time saved, decreased cost, socially distance | 37% felt an in-person visit would be better for child's clinical care, Inability of provider to perform a physical exam, tech issues (1/3), lack of personal connection |
| Mahmoud et al., 2022^65^ | Egypt | Ambulatory and Surgery | Telemedicine | To explore the suitability of a telemedicine intervention to overcome the social barriers imposed by the COVID-19 pandemic | Surveys | Mixed Methods | Video Calls and a Chat Program | Guardians | - Satisfaction: 92% of parents were satisfied | - Satisfaction: 8% of parents were dissatisfied  - Technical: Internet connectivity  - Engagement: Not convinced of online encounter; Appointment interfered with duties |
| Makkar et al, 2020^66^ | United States | NICU | Telemedicine | To evaluate the safety and efficacy of premature infant treatment managed by hybrid telemedicine versus conventional care | Survey Data | Observational (cohort/case study/cross-sectional/prospective/longitudinal) | Videoconferencing | Caregivers | Parental satisfaction with the use of hybrid telemedicine was particularly high, Families spent more time with their babies due to TM, the infants were able to stay at a local hospital without compromising the quality of care | Not applicable |
| Marques et al, 2022^67^ | United States | General Pediatric | Telemedicine | Aim to evaluate and compare patients' telemedicine and in-person experience for ambulatory encounters based on survey data throughout 2020, with particular focus on the influence of distance of the patients' home address from the medical facility. | Survey Data | General Quantitative- unspecified | Hybrid (virtual and/or in person) | Caregivers and Children/Youth | 81% recommend TH after doing it, Patients who lived farther had higher satisfaction scores regardless of visit type (p<0.01) | Not applicable |
| Mateus et al., 2023^68^ | Canada | Emergency Medicine | Telemedicine | Explored experiences of their child receiving care through telemedicine. | Interviews for parents | Mixed methods | Does not provide details on format but does say that intervention was a two-way audio-visual consultation | Parents | - Benefits: Parents felt the remote intervention aided in their child's transfer from being at a local hospital to a remote hospital.  - Child’s health: Parents felt their child had positive outcomes and there was no reported adverse outcomes.  - Relationship between provider and parent: Parents were unfamiliar with intervention but became confident and assured of the care once the intervention began. Parents were appreciative to being connected to a team of experts. | Not applicable |
| McCoy et al, 2022^69^ | United States | Otolaryngologist (Ear, nose, and throat) | Telemedicine | Assess satisfaction of otolaryngology outpatient visits during the pandemic between three study groups: those seen in telemedicine, those seen in-person during the telemedicine period, and those seen in-person before telemedicine was implemented. | Survey Data | Observational (cohort/case study/cross-sectional/prospective/longitudinal) | Videoconferencing | Caregivers | 69,5% would seek TM outpatient visit in the future, 81.4% believe that TM made it easier for their child to see a specialist, convenience, access to care, travel cost |  |
| McLardie-Hore et al., 2020^70^ | Australia | NICU | Telephone phone support | To evaluate a remote intervention to support mothers who are breastfeeding | Surveys | Mixed Methods | Telephone + In-person (Care delivered in the hospital) | Mothers | - Benefits: Peer support via phone created a safe environment, offered education related to breastfeeding, child development and caregiving, gave them emotional support  - Satisfaction: Many felt telephone support was helpful but only a small percentage felt neutral. | - Fit of Support: A small percentage of mothers felt the support was not suited to them due to difficulties of coordinating calls and feeling the calls added more stress to becoming a mother  - Satisfaction: Only 15% found telephone support was negative stating that they received limited advice, had difficulty making the call and not having anything to talk about. A small percentage (6%) felt the support resulted in negative self-perceptions (e.g., felt criticized). |
| McNally et al, 2022^71^ | United States | Neurodevelopmental Disabilities | Telehealth | Specifically sought to describe telehealth neurodevelopmental evaluations, examine associations between child characteristics and diagnostic factors, determine the impact of technology and family barriers, and report on clinician and caregiver satisfaction with telehealth evaluation. | Survey Data | General Quantitative- unspecified | Videoconferencing | Caregivers and Children/Youth | Presence of technology barriers was not associated with the ability to provide a telehealth diagnosis or the clinician’s dichotomized (i.e., certain or uncertain) ratings of diagnostic certainty, High across 19 domains of satisfaction, travel time savings | Tech issues |
| Meininger et al, 2022^72^ | Germany | Rehab (Physical, speech, occupational, cognitive-behavioural, neuropsychological, psychological support) | Other | The aim was to evaluate the implementation and especially the acceptance of a satisfaction with teletherapy in a large sample of patients of the outpatient unit for cognitive behavioral therapy at AKiP. | Survey Data | General Quantitative- unspecified | Videoconferencing | Caregivers | For more than 70% of all patients, teletherapy was accepted by the therapists, patients, and parents. 82% at least partially satisfied |  |
| Mena et al., 2022^73^ | Dominican Republic | Multidisciplinary Management | Telemedicine | To examine the use of telemedicine as a collaboration tool between multidisciplinary teams and to explore the experience of patients | Survey | Mixed Methods | Video Calls | Family | - Satisfaction: Most felt that the equipment worked well (93%), felt technology was not a barrier to communication (82%), felt heard (93%), felt their questions were answered (96%), and felt their privacy was protected (89%). All felt confident in the care they received. | - Satisfaction: About a third (32%) felt it was hard to obtain support within their own region. |
| Mimila et al., 2017^74^ | United States of America | Pediatrics | Telemedicine | To explore the experience of parents whose child receives preventative and developmental services | Survey and Interviews | Mixed Methods | Web-based tool | Parents | - Benefits attributed to parent coach: Felt coach was helpful, offered useful and socio-emotional support, did follow-ups/check-ups after appointment and was attentive to needs and made them feel comfortable.  - Benefits attributed to planner: Some felt planner provided useful information; Most preferred to use planner via personal phone.  - Benefits attributed to texting: Found texts were useful.  - Benefits attributed to care team: Felt team used time effectively.  - Relationship between parent and parent coach: Parents felt a positive and trusting relationship with coach | - Texts: A few parents did not have resources for texting (e.g., no phone, no data plan) or had their phone number changed |
| Mollen et al., 2019^75^ | United States of America | Emergency Medicine | Real-time Audiovisual Consultation | To describe caregiver perceptions of the decision to transfer his or her child to a pediatric emergency department (ED) and the potential use of telemedicine as an alternative to transfer | Not Known | Qualitative (Grounded Theory) | No intervention | Caregivers | - Preferences: Parents felt telemedicine was helpful but emphasized the importance of fitting telemedicine to the context (e.g., child’s condition, resources for staff/resources, care-related costs) | - Preferences: While parents felt telemedicine could be good, concerns with comfort, effective communication and fit of technology with care for child. |
| Moreno & Peck, 2020^76^ | United States of America | Otolaryngologist (Ear, nose, and throat) | Telehealth | To improve access to care with creation and implementation of a hospital-based discharge protocol and adoption of telehealth follow-up care for newly placed tracheostomy tubes. | Survey Data | General Quantitative- unspecified | Telephone Calls | Caregivers | Fully cooperative with process and expressed gratitude for remote follow-up care, open dialogue between caregiver and the provider | Only 2 patients met inclusion criteria |
| Netson et al, 2023^77^ | United States of America | Surgical Care | Telemedicine | To address the following: (i) explore the clinical pathways during the COVID-19 pandemic particularly exploring patient access and patient experiences, (ii) validate the non-inferiority of virtual visits (pre-operative) vs. in-person visit, (iii) explore ways to validate patient experience measures in order to capture patient experiences and (iv) ascertain if specific clinical diagnoses are a better fit for surgical decision-making using virtual visits. | Survey Data | General Quantitative- unspecified | Videoconferencing | Children/Youth | High patient satisfaction ratings among all aspects of care received - 90 % of participants rated that they would recommend the service to others. Same time with physician regardless of TM or in person. Virtual visits were non-inferior to traditional in-person visits as measured by patient surveys. Virtual appts more readily available, access to care improved slightly. |  |
| Nguyen et al, 2015^78^ | United States of America | Cardiothoracic | Other | Assess the usefulness of pediatric ECG tracings generated by the AliveCor device (Oklahoma City, OK) and to assess user satisfaction. | Survey Data | General Quantitative- unspecified | Telephone Calls | Children/Youth | 98% of the survey responses indicated that it was easy to obtain tracings, 93% showed interest in continued use of the device after the study period ended. | Some felt it was hard to do on active infants or toddlers, business hours only help. |
| Odeh et al, 2020^79^ | Jordan | Endocrinology | Telemedicine | To evaluate the effect of these measures  on paediatric patients with type 1 diabetes in terms of acute metabolic complications  and shortages in insulin and glucose measuring supplies. | Survey Data | General Quantitative- unspecified | Telephone Calls | Caregivers | 85.5% of participants said it was a smooth and positive experience. None of the patients were diagnosed with covid | Inability to compare the daily glucose readings prior and during the lockdown. |
| Olateju at al, 2022^80^ | United States of America | Endocrinology | Telemedicine | Add to current literature by describing caregiver perceptions on the acceptability of adolescent telemedicine visits in terms of  both perceived accessibility and satisfaction | Survey Data | Observational (cohort/case study/cross-sectional/prospective/longitudinal) | Telephone Calls, Hybrid (virtual and/or in person) | Caregivers | 82% said TM was easy to use, 90% believed their kid's needs were met, for many caregivers of adolescent patients, telemedicine is at least equally as acceptable and satisfactory as in-person medical visit | Low response to survey invitation |
| Onofri et al, 2021^81^ | Italy | Cardiothoracic | Telemedicine | To describe our experience with telemedicine (teleconsultation [TC] and telemonitoring of ventilator [TM]) in CMC on ventilation. | Observation | General Quantitative- unspecified | Videoconferencing | Children/Youth | Caregivers and clinicians were comfortable with the device and found it useful for data gathering, Fewer hospital days and reduced cost rate | Some tech issues |
| Phillips et al., 2021^82^ | Australia | Critical Care | Telehealth | To develop a telehealth burn clinic. | Individual Interviews | Qualitative (Phenomenology) | No intervention | Parents | - Benefits: Telehealth review clinic ensured continuity of care, connected families to experts who could assess and treat child’s burns, reduced travel costs and interruptions in family’s daily life  - Relationship between provider and parent: Families felt they had effective communication and felt included in the decision-making process of their child’s care | - Engagement: Telehealth clinic became challenging due to difficult discussions due to technical jargon expressed by provider, confusion about the roles of different providers.  - Technical: Rural and remote areas had connectivity issues and inability to obtain proper equipment; poor quality of images; difficulty in using equipment |
| Qubty et al, 2018^83^ | United States of America | Neurology | Telemedicine | Survey our patients about their experience with our  clinic’s telemedicine program to better understand telemedicine’s utility for families, and to  improve patient satisfaction and ultimately patient care. To understand how telemedicine is convenient, perceived to be cost-effective, and patient-centered | Survey Data | Observational (cohort/case study/cross-sectional/prospective/longitudinal) | Videoconferencing | Caregivers and Children/Youth | Preferred a TM follow-up to a clinic visit, lower no-show and late arrival frequency, Convenience, cost of travel saved, more time in school, on homework, or extracurriculars, Families saved on average $486 | Hard to examine patient over video, Suboptimal audio and video quality as well as connectivity issues on home internet |
| Rashid et al, 2021^84^ | Canada | Gastroenterology | Telehealth | Assess the feasibility and effectiveness of  delivering gluten-free diet (GFD) education remotely. | Survey Data | Experimental | Videoconferencing | Caregivers | Participant knowledge of gluten free diet increases significantly after an education session with dietician, Enhanced learning opportunities, improved access to education, greater flexibility, increased interaction and collaboration, cost and time savings | May feel overwhelmed by the length of a session and/or amount of info given, Internet connectivity issues, organizing sessions |
| Reid et al, 2021^85^ | Canada | Emergency Medicine | Other | To understand the feasibility, utilization rate, and satisfaction of the first Virtual Pediatric ED (V-PED) in Canada. Examine the feasibility and impact of virtual care as an adjunct to in-person  emergency care at the emergency department (ED). | Survey Data | Observational (cohort/case study/cross-sectional/prospective/longitudinal) | Hybrid (virtual and/or in person) | Children/Youth | High satisfaction with TM during post visit telephone interviews, Access to care increased, no risk of getting sick, addressed concerns and would use in the future, saved time and felt safer | Pediatric physical exam is impossible |
| Ruskin et al., 2023^86^ | Canada | Psychology & Physiotherapy | Virtual Multidisciplinary Treatment Care | To examine patient and caregiver satisfaction with virtual multidisciplinary treatment care for pediatric chronic pain during pandemic | Survey and Interviews | Mixed Methods | Video Calls | Children and their caregivers | - Satisfaction with equipment: Most families were satisfied with picture quality (100% for parents; 90% for children) and sound (100% for parents; 85% for children), had access to video conferencing technology (95% for parents; 85% for children) and did not have trouble accessing appointment links (85% for parents; 80% for children);  - Satisfaction with communication and rapport: Most families felt comfortable interacting with providers in virtual care (85% for parents; 80% for children), sharing sensitive information via a virtual platform (75% for parents; 65% for children)  - Satisfaction with clinical assessment and intervention: Most families felt confident in the providers’ ability to assess chronic pain needs (70% for parents; 60% for children), provide treatment via virtually compared to in-person (70% for parents; 75% for children), felt their questions were answered by their provider (95% for parents; 100% for children), received sufficient information (80% for parents; 75% for children), had sufficient time with their provider (95% for parents; 85% for children); felt respected by the provider (95% for parents; 95% for children) and had no privacy concerns (95% for parents; 100% for children) | - Engagement: Unable to read non-virtual cues; Lack of ability to focus on screen or screen fatigue; Feeling anxious or uncomfortable during virtual appointments; Felt sense of losing important information; Belief that learning rehabilitation exercises may be difficult in a virtual setting. In contrast, participants felt in-person appointments were comfortable, easier and promoting motivation  - Preferences: Many children preferred to have an in-person visit; If COVID-19 was not ongoing, many children and some caregivers preferred to have a virtual visit; If an in-person appointment was offered, some children and many parents wish for a virtual appointment; Children wanted the initial appointment to be in-person; In-person appointments should be at the beginning in order to transition easily to virtual platforms and establish rapport; Preference to have hybrid appointments overseen by an administrative virtual system; Administer treatment that can be tailored to patient’s needs.  - Satisfaction: A small percentage of parents and children reported feeling more uncomfortable communicating with providers via virtual platform compared to communicating with providers via in-person.  - Technical: Internet connectivity, audio and visual lags, device limitations |
| Severini et al, 2022^87^ | Brazil | Emergency Medicine | Telemedicine | Would this study work for future implementation in other public services in Brazil and developing countries? Aims to describe the implementation of a low-cost telemedicine service in a pediatric hospital in Brazil. | Survey Data | General Quantitative- unspecified | Videoconferencing | Caregivers and Children/Youth | Great satisfaction with guardians, Reduced stress of not being at risk of covid in hospital, possibility of remote diagnosis, provision of emergency care, saved cost, increased patient safety | Connection problem and difficulty with application |
| Shamsi et al., 2023^88^ | Iran | Neonatal Intensive Care Unit | Telehealth | To provide support to mothers so they can address their infants’ concern | Semi structured interviews | Content Analysis | Apps (WhatsApp and Telegram) | Mothers (Half of sample were hospitalized and half were discharged) | - Benefit: Mothers can exchange experiences with each other.  - Caregiver Outcome: Mothers enhanced their knowledge of caring for a preterm infant.  - Engagement: Mothers appreciated the communication with the health care team. | - Engagement: Mothers who were hospitalized were uncertain about the telehealth nurse. However, the discharged mothers felt telehealth nurses were supportive. |
| Sharma & Daniel, 2020^89^ | United Kingdom | Otolaryngologist (Ear, nose, and throat) | Telemedicine | Are telephone consultations appropriate for pediatric otorhinolaryngology patients? | Survey Data | General Quantitative- unspecified | Hybrid (virtual and/or in person) | Caregivers and Children/Youth | Patients reported that 29% would choose telephone consultation given the choice, 43% face to face, and 26% stated it would depend on the problem, effective triaging and redirection to appropriate pathways as required, reduced outpatient waiting lists, Reduced wait time, reduced travel and parking costs | There may not have been enough opportunity for the parent to fully process the pros and cons of the telephone conversation before offering feedback. Telephone contact could not be made with 21% of families despite booked appointments and multiple calls. All of the children with neck lumps required a face-to-face clinic appointment after the telephone consultation. |
| Shulman et al, 2023^90^ | United States of America | Pediatric Rehabilitation | Telehealth | To determine the differences in patient outcomes between a rehab given through a hybrid model and a traditional, in-person model. | Observation | Observational (cohort/case study/cross-sectional/prospective/longitudinal) | Hybrid (virtual and/or in person) | Children/Youth | 94% will use video clinics again, could easily hear and talk to the clinician, saved family time, was easy to use | One parent had connectivity issues |
| Sikka, 2023^91^ | India | Multiple specialties | Other | Assess how feasible teletherapy is, and to determine its efficiency over physical consultation and therapy sessions according to the parents of children with speech and language disorder. The present study aimed at exploring the parental perspective on teletherapy, for children having speech and language delay, during the covid-19 pandemic lockdown. | Survey Data | Observational (cohort/case study/cross-sectional/prospective/longitudinal) | Multiple/Mixed (ex. video and/or phone calls) | Caregivers and Children/Youth | 95% of parents reported improved motivation for speech-language therapy for their child due to teletherapy during the COVID-19 pandemic. Speech therapy able to continue TM, teletherapy helped work together to improve their child's language skills. 88% of parents believe face-to-face consultation is also required after some time. Cost-effective. | Gadgets have increased along with increased screen time which can have ill effects on children with speech and language delays. |
| Singh & Datta, 2020^92^ | United Kingdom | General Pediatric | Teleconsultation | Patient's perspectives of telephone consultations | Survey Data | Observational (cohort/case study/cross-sectional/prospective/longitudinal) | Multiple/Mixed (ex. video and/or phone calls) | Caregivers and Children/Youth | Decreased risk of infection, before the pandemic follow up wait was up to 1.5 years, but we were able to clear the backlog completely and to see children within the recommended time scale. No stress of driving, time saving, convenient, no parking fee, saves transport difficulties, no need to take time out of school | Can't examine the child physically and record their growth parameters. Language problems, virtual tech was too hard for some, feels more personal face to face |
| Sinha et al., 2019^93^ | United States of America | Orthopedics | Telemedicine | To assess patient satisfaction in using telecommunication for fracture care | Medical Charts and Surveys | Mixed Methods | Video Calls delivered on a tablet or a personal computer | Children and their parents | - Satisfaction: High ratings of telemedicine experience  - Preference: More than a third wish to have a teleconsultation. | - Engagement: Those who had an in-person appointment felt they could understand their provider compared to those who received a virtual appointment. |
| Smith et al., 2023^94^ | United States of America | Occupational therapy (Rehabilitation) | Telehealth | Examine parent satisfaction levels and experiences with the telehealth approach | Surveys and interviews | Mixed Methods | Video calls (Zoom) | Parents | -Benefits: Easy to use, useful, effective, reliable, satisfactory, flexibility in accommodating schedule changes, no need to plan for travel or accommodations (e.g., daycare), enables parents to be more involved and share in implementing strategies for their child.  -Engagement with providers: Enhanced parent-provider communication, relaxing environment | -Engagement: Two parents felt telehealth did not replace the 'in-person feel' of the service or enable parents to read body language. |
| Sprecher et al., 2023^95^ | United States of America | Emergency Medicine and Respiratory | Telehealth | To compare quality, safety and equity of access and use of virtual acute care vs. in-person acute care during pandemic and beyond | Administrative Data, Surveys and Focus Groups | Mixed Methods | Not applicable | Parents | - Treatment for child: Virtual visits were associated with a 13.2% decrease in antibiotic use. | - Engagement: Parents did not know how to assess the child’s symptoms and could not feel the presence of the provider.  - Technical: Some parents had concerns about accessing specific technology for the virtual appointment (e.g., having a smart phone, having a laptop with a camera) |
| Stagg et al., 2023^96^ | United States of America | Cardiac | Telemedicine | To explore how eHEALTH could improve communication between parents of premature infants and home health care nurses and enhance families' feeling of confidence in caring for their infant. | Clinical Data and Survey | Mixed Methods | Telephone + Email/Text + Logging onto a website portal + Parent must upload data onto a hospital specific app | Parents | - Child’s health: Virtual care alerted care team of 6 patients significant who had lowered clinical outcomes (e.g., decreased saturations, tachypnea, poor feeding) leading to therapeutic changes and avoiding emergency visits/hospitalizations; In 4 patients, virtual care helped to expediate cardiac care treatment; in 2 patients, virtual care resulted in additional consultations | - Technical: 1 incident of technical failure (e.g., pulse oximeter); 17% of caregivers reported poor audio |
| Stewart et al, 2022^97^ | United States of America | Ophthalmology | Telemedicine |  | Survey Data | Exploratory | Videoconferencing | Caregivers and Children/Youth | 98.5% of parents/guardians were satisfied with the quality of the telemedicine, 97% would use it again. Video helped doctors see their child's condition better, shorter wait period (1 month compared to 8 months), Telemedicine allowed the ophthalmologist to bypass a cumbersome referral system enabling better for patients | Equipment challenges, network issues reported. |
| Strand et al., 2020^98^ | Sweden | NICU | eHealth | To develop an eHEALTH device that supports the transition from hospital to home for patients with a preterm born child | Individual Interviews | Qualitative | Hybrid (Video and Text for Communication; Tablet is used to store and monitor child's health information) | Parents | - Caregiver outcome: Parents felt a sense of control and security in managing their child’s care (e.g., could upload information, receive information from provider)  - Benefits of eHealth app: App created a way to communicate with care team when needed, foster a sense of safety and reassurance, facilitate understanding using available features, manage visits and enable parents to share immediate questions via chat  - Benefits of IT System: IT system enabled communication between family and care team, communicate in multiple formats (e.g., video, text, pictures) and replaced some in-person visits | - Did not mention any |
| Sultan et al, 2020^99^ | United States of America | Orthopedics | Telemedicine | Evaluate the feasibility and satisfaction associated with virtual visit utilization in pediatric spinal deformity patients | No stated data collection | General Quantitative- unspecified | Telephone Calls | Caregivers and Children/Youth | Both groups satisfied with TM, Decrease patient costs for transportation | Lengthy time for providers to review patient data, telemedicine may not be covered by insurance |
| Taddei & Bulgheroni, 2020^100^ | Italy | Psychology | Telehealth | Describe the implementation of telehealth into clinical practice and to provide information about its feasibility and satisfaction of families | Survey Data | Exploratory | Videoconferencing | Caregivers and Children/Youth | Child received attention they needed. Caregivers were highly satisfied with video sessions as a means of receiving care. Save time and money | Caregivers felt the video sessions lacked empathy compared to in person. Language barrier, tech issues, difficulties to hear |
| Tan & Ganapathy, 2023^101^ | Singapore | Emergency Medicine | Telemedicine | Level of parents' satisfaction with the covid telemedicine consultation and determining children's ED reattendance or hospital admission within 10 days after the consultation. | Survey Data | Observational (cohort/case study/cross-sectional/prospective/longitudinal) | Videoconferencing | Caregivers and Children/Youth | High level of overall satisfaction, parents believed the virtual healthcare improved medical care for their child, all patients survived, improves medical care | Pulse oximeter was difficult to use at home for parents/caregivers, lack of physical examination, process for collection of medications wasn't convenient, need for strong internet connections/software/equipment |
| Trace et al., 2020^102^ | United Kingdom | Nephrology & Nutrition | Videoconsultation | To explore virtual dietetic consultations | Individual Interviews | Qualitative | Video Calls | Children and Parents | - Benefits: Video consultation enabled face-to-face conversation, deeper understanding of child’s progress; Reduction in travel; Staying safe at home  - Satisfaction: Video consultation felt personable, friendly and created a trusting space  - Preference: Parent opted for a platform that was convenient, incorporated scheduling and to use virtual care as a supplement to local care; Children wished for future video consultation particularly those who had severe chronic kidney disease | - Access: Connecting via email/telephone were only allowed for the parent  - Engagement: Could only engage with older but not younger children  - Technical: Visual-audio issues; Connectivity issues, privacy issues |
| Trivisano et al, 2020^103^ | Italy | Neurology | Telehealth | Explore children and parents' perspectives of using telehealth in caring for children with epilepsy. | Survey Data | Exploratory | Multiple/Mixed (ex. video and/or phone calls) | Caregivers and Children/Youth | Shorter waiting time. Convenience, tool for routine visits | 6% were unsatisfied/disappointed with the remote consultation due to internet connection (6.1%) or felt the remote consultation was inadequate for their concern (20.2%). Remote consultations would not be useful for neurologic assessments (60.7%) and psychological consultation (59.6%) |
| Tsai et al., 2023^104^ | Australia | Dentistry | Telehealth | Explore the experience, satisfactions and views on the feasibility of an oral health primary prevention telehealth service | Survey | Mixed Methods | Video calls | Parents | -Benefit: 56.8% of parents felt they had better access to care  - Caregiver Outcome: Of parents, 94.6% gained skills in helping their child to have health teeth, 91.9% felt they improved their child's diet and 97.3% felt confident to apply learned skills in life.  -Engagement: Of parents, 81.1% found it easy to communicate with the provider.  -Satisfaction: Of parents, 97.3% were satisfied overall with the intervention, 81.1% found the intervention to be acceptable, 89.2% wished to use this service again and 91.9% felt it was simple and easy to use.  - Comparison of groups: 30–39-year-old parent groups & parents who spoke English primarily in the home were more significantly likely to agree that telehealth was an acceptable way to receive care. (p = .048). English-speaking parents also were more significantly likely to use telehealth services again (p = 0.033). | -Platform: 37.8% of parents felt in-person would be more effective |
| Tschamper et al., 2018^105^ | Norway | Neurology | Videoconference | To explore parents' experiences of information exchange between tertiary health service and the child's multidisciplinary local support service using virtual conferencing | Focus Group Interviews | Qualitative | Video Calls | Parents | - Benefit: Video consultation enabled ongoing communications between parents and providers, promoted attentiveness due to exchange of information and visuals, created a safe space to talk about sensitive information; Families were not bothered by lack of eye contact  - Relationship between provider and parent: Parents felt confident and trusted child’s provider; Parents felt provider could advocate for child’s needs | - Engagement: Exchanging information led to objectification of child; Lack of eye contact with an unfamiliar person created a sense of discomfort when discussing issues; Potential for unexpected reactions to emerge  - Technology Familiarity: Lack of familiarity with platform made those feel less comfortable |
| von Sengbusch et al., 2020^106^ | Germany | Diabetes | Virtual Consultation | To explore how monthly video consultations supplemented with regular care can influence the clinical outcomes of children diagnosed with Type 1 diabetes | Clinical Data | Mixed Methods | Video Calls | Families (Children and their parents) | - Caregiver outcome: Improved maternal self-efficacy  - Child’s Health: Decreased clinical outcomes for child (e.g., lowered blood sugar levels), improved psychosocial outcomes and less hospital treatments  - Satisfaction: Satisfaction with care treatment | - Did not mention |
| von Sengbusch et al., 2021^107^ | Germany | Diabetes | Virtual Consultation | To examine parents' expectations of the perceived barriers and benefits of receiving 1 year of monthly video consultation + outpatient care for their child who has Type 1 diabetes | Interviews | Mixed Methods | Video Calls | Families (Children and their parents) | - Benefits: Less worry to travel, wait for provider, find accommodations for other children  - Caregiver Outcome: Felt more involved in caregiving duties of child (e.g., voiced their perspective with the provider, improve knowledge about diabetes); for anxious and insecure parents, those who received counseling, they felt emotionally secure about managing child’s care  - Engagement: Parents observed greater involvement of child in their care (e.g., increased interest during virtual appointments, better self-monitoring and self-management; Some parents initially had doubts about using virtual care but their confidence increased as they used the technology. | - Did not mention |
| von Sengbusch et al, 2022^108^ | Germany | Endocrinology | Telemedicine | Explore the clinical impacts of the monthly video consultations received by children and adolescents with Type 1 diabetes mellitus | Survey Data | Observational (cohort/case study/cross-sectional/prospective/longitudinal) | Videoconferencing | Caregivers and Children/Youth | Parents were positively satisfied with continuing treatment via TM care. Mean sensor glucose value and glucose management indicator slightly decreased. Significant improvement in parents satisfaction and their diabetes burden and distress |  |
| Wade et al, 2019^109^ | United States of America | Psychology | Telehealth | To understand the treatment modality preferences (online guided, in-person, self-guided) and its associations between preferences and drop-outs, adherence, satisfaction and efficacy | Survey Data | Exploratory | Videoconferencing | Children/Youth | Not applicable | According to parents and youth perspectives, therapy guided online sessions were not considered the most convenient treatment in comparison to self-guided online sessions. |
| Walijee et al, 2020^110^ | United States of America | Otolaryngologist (Ear, nose, and throat) | Teleconsultation | To explore the patient satisfaction and experiences of a nurse-led telephone follow-up service following surgery for sleep disorders | Survey Data | Observational (cohort/case study/cross-sectional/prospective/longitudinal) | Telephone Calls | Caregivers and Children/Youth | Discrete personalized care. Convenience, confidence, reassurance | (4%) preferred to talk to an ENT clinician or have an outpatient clinic visit vs. having a telephone consultation |
| Weaver et al, 2021^111^ | United States of America | General Pediatric | Telehealth | Explore the potential for a virtual bereaved parent support and the parents' perspectives of accepting technology and their experience of the group dynamics. | Survey Data | Experimental | Videoconferencing | Caregivers | Felt equipped to support their children in grief. Easy to learn, to use, felt it was acceptable. Benefitted from the program including communication, coping skills, peer support, education and an opportunity to express their grief | Only half of the parents completed all sessions. |
| Weaver et al, 2021^112^ | United States of America | General Pediatric | Telehealth | To explore the clinical and psychosocial outcomes of end-of-life pediatric patients who receive care from an adult-trained hospice team with scheduled telehealth support from a pediatric palliative care team; To explore the caregiver well-being over time | Survey Data | Exploratory | Videoconferencing | Caregivers and Children/Youth | Child's pain decreased over time while being in hospice care, parents able to recognize symptoms (fatigue, pain, appetite). Improved family functioning | Parents would under-recognize child's symptoms (sadness, worry) |
| Weber et al, 2021^113^ | United States of America | NICU | Telehealth | To explore parents' perspective of the effects of using a video camera and an increase in babies receiving breastmilk | Survey Data | Exploratory | Videoconferencing | Caregivers | Parents felt involved in their child's care, more plans to breastfeed they were more likely to receive breast milk at discharge. | Lack of availability of cameras to be attached to baby's bedside. |
| Williams, 2021^114^ | Australia | Endocrinology | Telehealth | To explore the outcomes of the hospital's pediatric diabetic telehealth service | Mixed data collection (interviews + observation) | Exploratory | Videoconferencing | Children/Youth | Parents felt good support was given to them and their child. convenience, no need to travel, not taking time off work or school, no tech issues. |  |
| Yen et al, 2020^115^ | United States of America | Psychology | Other | To explore the feasibility, acceptability and clinical outcomes of piloting a suicidal program for adolescents (STEP) vs. an enhanced treatment program | Survey Data | Experimental | Multiple/Mixed (ex. video and/or phone calls) | Caregivers and Children/Youth | There was a significant effect on treatment condition (favoring STEP) on depression and impairment. STEP participants had a high rate of acceptability from both parents and teens (85-100%). | A small percentage (19%) of the STEP (suicidal program for adolescents) participants had a suicide event |
| Young et al, 2019^116^ | United States of America | Multiple specialties | Telemedicine | To analyze the impact of pediatric telemedicine on wait times, visit durations and schedules | Survey Data | Exploratory | Videoconferencing | Children/Youth | 87% (Out of 209 surveys) of patients were very likely to schedule a virtual visit in the future. Quality care, improved access to care. Patients rated their virtual visits higher than those for in-person visits. Wait times were smaller compared to in person, convenience, increased efficiency. | Some tech issues (less than 20%) but were resolved in real time |
| Zayde et al, 2022^117^ | United States of America | Psychology | Telehealth | To assess clinical outcomes and treatment acceptability for the telehealth group parenting program for parents whose child was registered in an outpatient child mental health clinic. | Survey Data | Experimental | Videoconferencing | Caregivers and Children/Youth | Significant effect on depressive and anxiety symptoms. Felt sense of being part of a group, felt understood, accepted, and respected by the group/leader | Attendance dropped minimally over TH. Difficulty with zoom tech, confidentiality, data security. |

References

1. Akobeng AK, O’Leary N, Vail A, Brown N, Widiatmoko D, Fagbemi A, Thomas AG. Telephone Consultation as a Substitute for Routine Out-patient Face-to-face Consultation for Children With Inflammatory Bowel Disease: Randomised Controlled Trial and Economic Evaluation. EbioMedicine. (2015) 2(9):1251-1256. doi:10.1016/j.ebiom.2015.08.011
2. Alghamdi SA. Parent perceptions regarding virtual pediatric dental clinics during COVID-19 pandemic: a cross-sectional study. Peer J. (2023) 11:1-14. Doi:10.7717/peerj.15289
3. Alzahrani AM, Magliah SF, Turkistani HA, Abulaban BA, Sabban MF, Mashat MA, Al Shaikh AM. Perception of primary caregiver toward virtual pediatric clinics for type 1 diabetes mellitus during COVID-19 pandemic in Jeddah, Saudi Arabia: A cross-sectional study. Ann Med Surg. (2022) 81:1-6. Doi:10.1016/j.amsu.2022.104550
4. Assenza, C, Catania H, Antenore C, Gobbetti T, Gentili P, Paolucci S, Morelli D. Continuity of Care During COVID-19 Lockdown: A Survey on Stakeholders' Experience with Telerehabilitation. Front Neurol. (2021) 11:1-10. doi:10.3389/fneu.2020.617276
5. Bales NJ, Perera DC, Foerster R, Poirier L, Ducis K. (2023). Analysis of a novel virtual pediatric concussion clinic in a rural setting. Childs Nerv Syst*.* (2023) 1-7. doi:10.1007/s00381-023-06231-5
6. Bate NJ, Xu SC, Pacilli M, Roberts LJ, Kimber C, Nataraja RM. (2021). Effect of the COVID-19 induced phase of massive telehealth uptake on end-user satisfaction. Intern Med J. (2021) 51(2):206-214. doi:10.1111/imj.15222
7. Bell S, Karamchandani U, Malcolmson K, Moosajee M. Acceptability of telegenetics for families with genetic eye diseases. Genes. (2021) 12(2):1-9. doi:10.3390/genes12020276
8. Bianciardi Valassina MF, Bella S, Murgia F, Carestia A, Prosseda E. Telemedicine in pediatric wound care. Clin Ter. 2016;167(1 Supplement), 21-23. doi: 10.7417/T.2016.1915
9. Blagdon A, Smith D, Bramfield T, Soraisham A, Mehrem AA. Evaluation of family and staff experiences with virtual rounding and bedside presence in a tertiary neonatal intensive care unit during the COVID-19 pandemic. J Telemed Telecare. (2022) 1-15. doi:10.1177/1357633X221081294
10. Brewster RCL, Zhang J, Stewart M, Kaur R, Arellano M, Bourgeois F. A Prescription for Internet: Feasibility of a Tablet Loaner Program to Address Digital Health Inequities. Appl Clin Inform. (2023) 14(2):273-278. doi:10.1055/a-2016-7417
11. Brothwood PL, Baudinet J, Stewart CS, Simic M. Moving online: young people and parents' experiences of adolescent eating disorder day programme treatment during the COVID-19 pandemic. J Eat Disord. (2021) 9(1):1-10. doi:10.1186/s40337-021-00418-4
12. Bullock DR, Vehe RK, Zhang L, Correll CK. Telemedicine and other care models in pediatric rheumatology: An exploratory study of parents' perceptions of barriers to care and care preferences. Pediatr Rheumatol. (2017) 15(1):1-8. doi:10.1186/s12969-017-0184-y
13. Carretier E, Bastide M, Lachal J, Moro MR. Evaluation of the rapid implementation of telehealth during the COVID-19 pandemic: a qualitative study among adolescents and their parents. Eur Child Adolesc Psychiatry. (2022) 32:963-973. doi:10.1007/s00787-022-02108-1
14. Caruso CG, Warren JB, Carney PA. Parent experiences of a remote patient monitoring program enabling early discharge from the neonatal intensive care unit with nasogastric tube feeding. J Neonatal Perinatal Med. (2023) 16:301-309. doi:10.3233/NPM-221181
15. Castro MJ, Rodriguez RJ, Hudson B, Weersing VR, Kipke M, Peterson BS, West AE. Delivery of cognitive behavioral therapy with diverse, underresourced youth using telehealth: Advancing equity through consumer perspectives. Evid Based Pract Child Adolesc Ment Health. (2022) 8(2):206-220. doi:10.1080/23794925.2022.2062687
16. Chalmers JA, Sansom-Daly UM, Patterson P, McCowage G, Anazodo A. Psychosocial assessment using telehealth in adolescents and young adults with cancer: A partially randomized patient preference pilot study. J Med Internet Res. (2018) 7(8):1-25. doi:10.2196/resprot.8886
17. Chan EYH, Liu MS, Or PC, Ma ALT. Outcomes and perception of cloud-based remote patient monitoring in children receiving automated peritoneal dialysis: a prospective study. Pediatr Nephrol. (2022) 38:2171-2178. doi:10.1007/s00467-022-05828-3
18. Charnell AM, Hannon E, Burke D, Iredale MR, Sutcliffe JR. Virtual consultations: delivering outpatient clinics in paediatric surgery during the COVID-19 pandemic. Ann Pediatr Surg. (2020) 16(1):1-5. doi:10.1186/s43159-020-00060-w
19. Cockrell H, Wayne D, Wandell G, Wang X, Greenberg SLM, Kieran K, Dick A, Bonilla-Velez J. Understanding hispanic patient satisfaction with telehealth during COVID-19. J Pediatr Surg. (2022) 58(9):1783-1788. doi:10.1016/j.pedsurg.2022.12.006
20. Costa-Cordella S, Carmona KB, Contreras ND, Cano AG, Bonta CM, Grasso-Cladera A. Digital adaptation of group activities in an outpatient center of a children’s hospital: Adolescent participants’ experiences. Medwave. (2023) 23(4):1-10. doi:10.5867/medwave/2023.01.25686
21. Darr A, Senior A, Argyriou K, Limbrick J, Nie H, Kantczak A, Stephenson K, Parmar A, Grainger J. The impact of the coronavirus (COVID-19) pandemic on elective paediatric otolaryngology outpatient services – An analysis of virtual outpatient clinics in a tertiary referral centre using the modified paediatric otolaryngology telemedicine satisfaction survey (POTSS). Int J Pediatr Otorhinolaryngol. (2017) 138:1-8. doi:10.1016/j.ijporl.2020.110383
22. Davis J, Gordon R, Hammond A, Perkins R, Flanagan F, Rabinowitz E, Simoneau T, Sawicki GS. Rapid Implementation of Telehealth Services in a Pediatric Pulmonary Clinic During COVID-19. Pediatr. (2021) 148(1):1-9. doi:10.1542/peds.2020-030494
23. Dempsey CM, Serino-Cipoletta JM, Marinaccio BD, O'Malley KA, Goldberg NE, Dolan CM, Parker-Hartigan L, Williams LS, Vessey JA. Determining factors that influence parents' perceptions of telehealth provided in a pediatric gastroenterological practice: A quality improvement project. J Pediatr Nurs. (2022) 62:36-42. doi:10.1016/j.pedn.2021.11.023
24. Doerdelman J, Frielitz FS, Lange K, Meinsen T, Reimers S, Ottersberg T, Katalinic A, Hiort O, Von Sengbusch S. Video Consultation for Parents with a Child Newly Diagnosed with Type 1 Diabetes: A Qualitative Study. Exp Clin Endocrinol Diabetes. (2022) 130(8):519-524. doi:10.1055/a-1655-5471
25. Edwards LM, Parry M. (2022). Telephone consultations to manage paediatric outpatient clinics during the COVID-19 pandemic: a service evaluation. Ir J Med Sci. (2022) 191(3):977-983. doi:10.1007/s11845-021-02672-6
26. Elbin RJ, Stephenson K, Lipinski D, Maxey K, Womble MN, Reynolds E, Covert K, Kontos AP. In-Person Versus Telehealth for Concussion Clinical Care in Adolescents: A Pilot Study of Therapeutic Alliance and Patient Satisfaction. J Head Trauma Rehab. (2022) 37(4):213-219. doi:10.1097/HTR.0000000000000707
27. Finkelstein JB, Cahill D, Young K, Humphrey K, Campbell CS, Nelson CP, Gupta A, Estrada CR. Telemedicine for Pediatric Urological Postoperative Care is Safe, Convenient and Economical. J Urol. (2020) 204(1):144-148. doi:10.1097/JU.0000000000000750
28. Finnegan R, Flynn A, Flanagan O. Exploring parental experiences of virtual paediatric neurodevelopmental consultations. Ir J Med Sci. (2022) 191(2):807-808. doi:10.1007/s11845-021-02583-6
29. Fletcher SE, Tsang VWL. The era of virtual care: Perspectives of youth on virtual appointments in COVID-19 and beyond. Paediatr Child Health. (2021) 26(4):210-213. doi:10.1093/pch/pxaa138
30. Fortini S, Espeche A, Caraballo R. Telemedicine and epilepsy: A patient satisfaction survey of a pediatric remote care program. Epilepsy Res. (2020) 165:1-5. doi:10.1016/j.eplepsyres.2020.106370
31. Foster CC, Macy ML, Simon N, Stephen R, Lehnig K, Bohling K, Schinasi DA. Emergency Care Connect: Extending Pediatric Emergency Care Expertise to General Emergency Departments Through Telemedicine. Acad Pediatr. (2020) 20(5):577-584. doi:10.1016/j.acap.2020.02.028
32. Frye WS, Gardner L, Mateus JS. Utilising telemental health in a paediatric outpatient psychology clinic: Therapeutic alliance and outcomes. Couns Psychother Res. (2022) 22(2):322-330. doi:10.1002/capr.12450
33. Gan Z, Lee SY, Weiss DA, Van Batavia J, Siu S, Frazier J, Zderic SA, Shukla AR, Srinivasan AK, Kolon TF, Zaontz MR, Canning DA, Long CJ. Single institution experience with telemedicine for pediatric urology outpatient visits: Adapting to COVID-19 restrictions, patient satisfaction, and future utilization. J Pediatr Urol. (2021) 17(4):480.e1-480.e7. doi:10.1016/j.jpurol.2021.05.012
34. Garne Holm, K, Brodsgaard A, Zachariassen G, Smith AC, Clemensen J. Parent perspectives of neonatal tele-homecare: A qualitative study. J Telemed Telecare. (2019) 25(4):221-229. doi:10.1177/1357633X18765059
35. Gefen N, Steinhart S, Beeri M, Weiss PL. Lessons learned during a naturalistic study of online treatment for pediatric rehabilitation. Int J Environ Res Public Health. (2021) 18:1-17. doi:10.3390/ijerph18126659
36. Giuseppe DB, Giuseppina N, Desiree S, Angela S, Maurizio G, Perrone S. Improving Care in Neonatal Intensive Units During the COVID-19 Pandemic: A Survey on Electronic Health Communication. J Intensive Care Med. (2022) 37(5):671-678. doi:10.1177/08850666211055058
37. Gund A, Sjöqvist BA, Wigert H, Hentz E. A randomized controlled study about the use of eHealth in the home health care of premature infants. BMC Med Inform Decis Mak. (2013) 13(1):1-11. doi:10.1186/1472-6947-13-22
38. Guttmann-Bauman I, Kono J, Lin AL, Ramsey KL, Boston BA. Use of Telehealth Videoconferencing in Pediatric Type 1 Diabetes in Oregon. Telemed J E Health. (2018) 24(1):86-88. doi:10.1089/tmj.2017.0072
39. Hale AE, Bujoreanu S, LaVigne TW, Coakley R. Rapid mobilization of an evidence-based psychological intervention for pediatric pain during COVID-19: The development and deployment of the comfort ability program virtual intervention (CAP-V). Children. (2023) 10:1-14. doi:10.3390/children10091523
40. Hallford HG, Szyld E, McCoy M, Makkar A. A 360 Evaluation of Neonatal Care Quality at a Level II Neonatal Intensive Care Unit when Delivered Using a Hybrid Telemedicine Service. Am J Perinatol. (2022) Nov 10:1-8. doi:10.1055/a-1932-9921
41. Heath J, Dancel R, Stephens JR. Postdischarge phone calls after pediatric hospitalization: An observational study. Hosp Pediatr. (2015) 5(5):241-248. doi:10.1542/hpeds.2014-0069
42. Hendra K, Neemuchwala F, Chan M, Ly NP, Gibb ER. Patient and Provider Experience With Cystic Fibrosis Telemedicine Clinic. Front Pediatr. (2021) 9:1-7. doi:10.3389/fped.2021.784692
43. Hiscock H, Pelly R, Hua X, West S, Tucker D, Raymundo CM, Dalziel K. Survey of paediatric telehealth benefits from the caregiver perspective. Aust Health Rev. (2022) 46(2):197-203. doi: 10.1071/AH21036
44. Hoi KK, Brazina SA, Kolar-Anderson R, Zopf DA, Bohm LA. A Longitudinal Comparison of Telemedicine Versus In-Person Otolaryngology Clinic Efficiency and Patient Satisfaction During COVID-19. Ann Otol Rhinol Laryngol. (2022) 131(11):1177-1184. doi:10.1177/00034894211055349
45. Holzman SA, Davis-Dao CA, Khoury AE, Fortier MA, Kain, NZ. Telemedicine and patient satisfaction during the COVID-19 pandemic: A case-control study of outpatient pediatric urology patients. J Child Health Care. (2021) 27(3):351-359. Doi:10.1177/13674935211058272
46. Hoyt-Austin AE, Miller IT, Kuhn-Riordon KM, Rosenthal JL, Chantry CJ, Marcin JP, Hoffman KR, Kair LR. Bonding, Relaxation, Separation, and Connection: Expressing Human Milk While Videoconferencing with the Hospitalized Premature Infant. Breastfeed Med. (2022) 17(8):653-659. doi:10.1089/bfm.2021.0214
47. Huscsava MM, Scharinger C, Plener PL, Kothgassner OD. The world somehow stopped moving: Impact of the COVID-19 pandemic on adolescent psychiatric outpatients and the implementation of teletherapy. Child and Adolesc Ment Health. (2022) 27(3):232-237. doi:10.1111.camh.12481
48. Hylén M, Nilsson S, Kristensson-Hallstrὂm I, Kristjánsdóttir G, Stenstrὂm Vilhjálmsson R. Access to health care perceived by parents caring for their child at home supported by eHealth – a directed approach introducing aperture. BMC Health Serv Res. (2022) 22:1-11. doi:10.1186/s12813-022-08398-0
49. Jones E, Kurman J, Delia E, Crockett J, Peterson R, Thames J, Salorio C, Kalb L, Jacobson L, Stone J, Zabel TA. Parent satisfaction with outpatient telemedicine services during the COVID-19 pandemic: A repeated cross-sectional study. Front Pediatr. (2022) 10:1-9. doi:10.3389/fped.2022.908337
50. Joseph HB, Kuppusamy S, Mahalik SK, Shetty AP, Das K. Telemedicine – a boon to parents of children with health care needs during COVID-19 pandemic: A qualitative study from India. Turk Arch Pediatr. (2022) 57(5):526-531. doi:10.5152/TurkArchPediatr.2022.22046
51. Jury SC, Walker AM, Kornberg AJ. The introduction of web-based video-consultation in a paediatric acute care setting. J Telemed Telecare. (2013) 19(7):383-387. Doi:10.1177/1357633X13506530
52. Kelly SL, Steinberg EA, Suplee A, Upshaw NC, Campbell KR, Thomas JF, Buchanan CL. Implementing a Home-Based Telehealth Group Adherence Intervention with Adolescent Transplant Recipients. Telemed J E Health. (2019) 25(11):1040-1048. doi:10.1089/tmj.2018.0164
53. Khoury M, Phillips DB, Wood PW, Mott WR, Stickland MK, Boulanger P, Rempel GR, Conway J, Mackie, AS, Khoo NS. Cardiac rehabilitation in the paediatric Fontan population: development of a home-based high-intensity interval training programme. Cardiol Young. (2020) 30:1409-1416. doi:10.1017/sS1047951120002097
54. Kilipiris EG, Horn F, Kolnikova M, Ochoa JV, Matuskova O, Jelovac D, Stebel A. Parental satisfaction from telemedicine in the follow-up of children operated for craniosynostosis during COVID-19 pandemic. Cleft Palate J. (2023) 60(5):562-568. doi:10.1177/10556656221074214
55. Kolb CM, Born K, Banker K, Barth PC, Aaronson NL. Improving attendance and patient experiences during the expansion of a telehealth-based pediatric otolaryngology practice. Otolaryngol Head Neck Surg. (2021) 164(5):952-958. doi:10.1177/0194599820965917
56. Kramer JL, De Asis K. Osteopathic interventions via telehealth in a pediatric population: a retrospective case series. J Osteopath Med. (2021) 121(11):857-861. Doi:10.151/jom-2021-0124
57. Lakshin G, Banek S, Keese D, Rolle U, Schmedding A. Telemedicine in the pediatric surgery in Germany during the COVID-19 pandemic. Pediatr Surg Int. (2021) 37(3):389-395. doi:10.1007/s00383-020-04822-w
58. Lanzarin CMV, von Wangenheim A, Rejane-Heim TC, Nascimento FS, Wagner HM, Abel HS, Junior JDDS, Xikota JC. Teleconsultations at a Pediatrics Outpatient Service in COVID-19 Pandemic: First Results. Telemed J E Health. (2021) 27(11):1311-1316. doi:10.1089/tmj.2020.0471
59. Lawrence J, Measey MA, Hoq M, Hiscock H, Rhodes A. Virtual health care for children: Parental willingness to adopt virtual health-care technologies. J Paediatr Child Health. (2022) 58(8):1323-1329. doi:10.1111/jpc.15974
60. Lee S, Dick BD, Jordan A, McMurty CM. A Parent-Targeted Group Intervention for Pediatric Pain Delivered In-Person or Virtually: Feasibility, Acceptability, and Effectiveness. Clin J Pain. (2023) 39(3):127-137. doi:10.1097/AJP.0000000000001100
61. Lindkvist RM, Sjostrom-Strand A, Landgren K, Johnsson BA, Stenstrom P, Hallstrom IK. "In a Way We Took the Hospital Home"-A Descriptive Mixed-Methods Study of Parents' Usage and Experiences of eHealth for Self-Management after Hospital Discharge Due to Pediatric Surgery or Preterm Birth. Int J Environ res Public Health. (2021) 18:1-13. doi:10.3390/ijerph18126480
62. Lo WB, Herbert K, Rodrigues D. Clinical effectiveness of and family experience with telephone consultation in a regional pediatric neurosurgery center in the United Kingdom, Journal of neurosurgery. Pediatr. (2021) 28(4):483-489. doi:10.3171/2021.3.PEDS20862
63. López Seguí F, Batlle Boada A, García García JJ, López Ulldemolins A, Achotegui Del Arco A, Adroher Mas C, García Cuyàs F. Families' degree of satisfaction with pediatric telehomecare: Interventional prospective pilot study in Catalonia. JMIR Pediatr Parent. (2020) 3(1):1-8. Doi:10.2196/17517
64. Love M, Hunter AK, Lam G, Muir LV, Lin HC. Patient satisfaction and perceived quality of care with telemedicine in a pediatric gastroenterology clinic. Pediatr Rep. (2022) 14(2):181-189. doi:10.3390/pediatric14020025
65. Mahmoud MA, Daboos M, Gouda S, Othman A, Abdelmaboud M, Hussein ME, Akl M. Telemedicine (virtual clinic) effectively delivers the required healthcare service for pediatric ambulatory surgical patients during the current era of COVID-19 pandemic: A mixed descriptive study. J Pediatr Surg. (2022) 57:630-636. doi:10.1016/j.jpedsurg.2021.11.018
66. Makkar A, McCoy M, Hallford G, Foulks A, Anderson M, Milam J, Wehrer M, Doerfler E, Szyld E. Evaluation of neonatal services provided in a level II NICU utilizing hybrid telemedicine: A prospective study. Telemed J E Health. 2020;26(2), 176-183. doi:10.1089/tmj.2018.0262
67. Marques S, Cruz JAW, da Cunha MAVC, Tuon FF, de Moraes TP, Zdziarski AD, Bomher ST, Donnelly LF, Capasso R. Patient and family experience with telemedicine and in-person pediatric and obstetric ambulatory encounters throughout 2020, during the COVID-19 epidemic: the distance effect. BMC Health Serv Res. (2022) 22(1):1-8. doi:10.1186/s12913-022-08037-8
68. Mateus LA, Law MP, Khowaja AR, Orlando E, Pace A, Roy M, Sulowski C. Examining perceptions of a telemedicine network for pediatric emergency medicine: A mixed-methods pilot study. Front Digit Health. (2023) 5:1-8. doi:10.3389/fdgth.2023.1181059
69. McCoy J, Shaffer J, Amber D, Dohar JE. Pediatric otolaryngology telemedicine amid a pandemic - And beyond. Int J Pediatr Otorhinolaryngol. (2022) 153:1-7. doi:10.1049/j.ijporl.2021.111014
70. McLardie-Hore FE, McLachlan HL, Shafiei T, Forster DA. (2020). Proactive telephone-based peer support for breastfeeding: a cross-sectional survey of women's experiences of receiving support in the RUBY randomised controlled trial. BMJ Open. (2020) 10:1-11. doi:10.1136/bmjopen-202-040412
71. McNally Keehn R, Enneking B, James C, Tang Q, Rouse M, Hines E, Raches C, Etling A. Telehealth evaluation of pediatric neurodevelopmental disabilities during the COVID-19 pandemic: Clinician and caregiver perspectives. J Dev Behav Pediatr. (2022) 43(5):262-272. doi:10.1097.DBP.0000000000001043
72. Meininger L, Adam J, von Wirth E, Viefhaus P, Woitecki K, Walter D, Döpfner M. Cognitive-behavioral teletherapy for children and adolescents with mental disorders and their families during the COVID-19 pandemic: a survey on acceptance and satisfaction. Child Adolesc Psychiatry Ment Health. (2022) 16(1):1-11. Doi:10.1189/s13034-022-00494-7
73. Mena R, Mendoza E, Mangano FT, Henrickson M, Scott M, DeFoor WR, Little KJ, Ruschman J, Prada CE. International Pediatric Multidisciplinary Management Using Telemedicine to Promote Equitable Care. Telemed J E Health. (2022) 29(5):674-685. doi:10.1089/tmj.2022.0165
74. Mimila NA, Chung PJ, Elliott MN, Bethell CD, Chacon S, Biely C, Contreras S, Chavis T, Bruno Y, Moss T, Coker TR. Well-Child Care Redesign: A Mixed Methods Analysis of Parent Experiences in the PARENT Trial. Acad Pediatr. (2017) 17(7):747-754. doi:10.1016/j.acap.2017.02.004
75. Mollen CJ, Henien M, Jacobs LM, Myers S. Parent Perceptions on Transfers to Pediatric Emergency Departments and the Role of Telemedicine. Pediatr Emerg Care. (2019) 35(3):180-184. doi:10.1097/pec.000000000000957
76. Moreno L, Peck JL. Nurse Practitioner-Led Telehealth to Improve Outpatient Pediatric Tracheostomy Management in South Texas. J Pediatr Health Care. (2020) 34(3):246-255. doi:10.1016/j.pedhc.2019.11.008
77. Netson RA, Miller S, Incorvia J, Shah A, Estrada CR, Toomey SL, Taghinia AH. Patient experience with virtual preoperative consultations in pediatric surgical specialties. J Pediatr Surg. (2023) 58(9):1776-1782. doi:10.1016/j.jpedsurg.2022.12.027
78. Nguyen HH, Van Hare GF, Rudokas M, Bowman T, Silva JNA. SPEAR trial: Smartphone pediatric electrocardiogram trial. PloS One. (2015) 10(8):1-9. Doi:10.1371/journal.pone.0136256
79. Odeh R, Gharaibeh L, Daher A, Kussad S, Alassaf A. Caring for a child with type 1 diabetes during COVID-19 lockdown in a developing country: Challenges and parents' perspectives on the use of telemedicine. Diabetes Res Clin Pract. (2020) 168:1-7. doi:10.1016/j.diabres.2020.108393
80. Olateju A, Cervantes M, Dowshen N. Kuhns LM, Dhar CP. Acceptability of Telemedicine Among Parents of Adolescent Patients in an Adolescent Clinic: Cross-sectional Survey Study. JMIR Pediatr Parent. (2022) 5(4):1-7. doi:10.2196/39704
81. Onofri A, Pavone M, De Santis S, Verrillo E, Caggiano S, Ullmann N, Cutrera R. Telemedicine in children with medical complexity on home ventilation during the COVID‐19 pandemic, Pediatr Pulmonol. (2021) 56(6):1395-1400. doi: 10.1002/ppul.25289
82. Phillips D, Matheson L, Pain T, Kingston GA. Evaluation of an occupational therapy led Paediatric Burns Telehealth Review Clinic: exploring the experience of family/carers and clinicians. Rural Remote Health. (2022) 22(1):1-8. doi:10.22605/RRH6887
83. Qubty W, Patniyot I, Gelfand A. Telemedicine in a pediatric headache clinic. Neurology. (2018) 90(19):E1702-E1705. doi:10.1212/WNL.0000000000005482
84. Rashid M, Haskett J, Parkinson McGraw L, Noble A, van Limbergen J, Otley A. Teaching Families of Children with Celiac Disease about Gluten-Free Diet Using Distributed Education: a Pilot Study. Can J Diet Pract Res. (2021) 82(1):38-40. doi:10.3148/cjdpr-2020-021
85. Reid S, Bhatt M, Zemek R, Tse S. Virtual care in the pediatric emergency department: a new way of doing business? CJEM. (2021) 23(1):80-84. doi:10.1007/s43678-020-00048-w
86. Ruskin D, Tremblay M, Szczech K, Rosenbloom BN, Mesaroli G, Sun N, D'Alessandro L. Virtual multidisciplinary pain treatment: Experiences and feedback from children with chronic pain and their caregivers. Physiother Theory Pract. (2023) 1-22. doi:10.1080/09593985.2023.2171750
87. Severini RDSG, Oliveira PCD, Couto TB, Simon Junior H, Andrade APMD, Nanbu DY, Farhat SCL, Schvartsman C. Fast, cheap and feasible: Implementation of pediatric telemedicine in a public hospital during the Covid-19 pandemic. J Pediatr (Rio J). (2022) 98(2):183-189. doi:10.1016/j.jped.2021.05.007
88. Shamsi A, Namnabati M, Ehteshami A, Zandi H. Telehealth experiences of mothers of hospitalized and discharged preterm infants in Islamic Republic of Iran. East Mediterr Health J. (2023) 29(5):309-316. doi:10.26719/emhj.23.055
89. Sharma S, Daniel M. Telemedicine in paediatric otorhinolaryngology: Lessons learnt from remote encounters during the Covid19 pandemic and implications for future practice. Int J Pediatr Otorhinolaryngol. (2020) 139:1-6. doi:10.1016/j.ijporl.2020.110411
90. Shulman J, Conroy C, Bento S, Bryant G, Jervis K, Sethna NF. Pediatric pain rehabilitation during the COVID-19 pandemic: exploring the effectiveness of a hybrid intensive interdisciplinary pain treatment model. Disabil Rehabil. (2023) 45(19):3079-3086. doi:10.1080/09638288.2022.2125083
91. Sikka K. Parent's perspective on teletherapy of pediatric population with speech and language disorder during Covid-19 lockdown in India. Indian J Otolaryngol Head Neck Surg. (2023) 75(1):14-20. doi:10.1007/s12070-022-03310-y
92. Singh N, Datta M. Single-centre telephone survey on patients' perspectives regarding remote paediatric outpatient consultations in a district general hospital. BMJ Paediatr. (2020) 4(1):1-2. doi:10.1136/bmjpo-2020-000885
93. Sinha N, Cornell M, Wheatley B, Munley N, Seeley M. Looking Through a Different Lens: Patient Satisfaction With Telemedicine in Delivering Pediatric Fracture Care. J Am Acad Orthop Surg Glob Res Rev. (2019) 3(9):1-6. doi: 10.5435/JAAOSGlobal-D-19-00100
94. Smith SL, Vaquerano J, Humphreys BP, Aytur SA. Parent satisfaction with a telehealth parent coaching intervention to support family participation. OTJR. (2023) 43(3):531-539. doi:10.1177/15394492231164942
95. Sprecher E, Conroy K, Krupa J, Shah S, Chi GW, Graham D, Starmer AJ. A Mixed-Methods Assessment of Coronavirus Disease of 2019-Era Telehealth Acute Care Visits in the Medical Home. J Pediatr. (2023) 255:121-127. doi:10.1016/j.jpeds.2022.10.036
96. Stagg A, Giglia TM, Gardner MM, Offit BF, Fuller KM, Natarajan SS, Hehir DA, Szwast AL, Rome JJ, Ravishankar C, Laskin BL, Preminger TJ. Initial Experience with Telemedicine for Interstage Monitoring in Infants with Palliated Congenital Heart Disease. Pediatr Cardiol. (2023) 44(1):196-203. doi:10.1007/s00246-022-02993-y
97. Stewart C, Coffey-Standoval J, Souverein EA, Ho TC, Lee TC, Nallasamy S. Patient and Provider Experience in Real-Time Telemedicine Consultations for Pediatric Ophthalmology. Clin Ophthalmol. (2022) 16:2943-2953. doi:10.2147/OPTH.S374811
98. Strand AS, Johnsson B, Hena M, Magnusson B, Hallstrom I.K. Developing eHealth in neonatal care to enhance parents' self-management. Scan J Caring Sci. (2022) 36(4):969-977. doi:10.1111/scs.12994
99. Sultan AA, Acuna AJ, Samuel LT, Rabin JM, Grits D, Gurd DP, Kuivila TE, Goodwin RC. Utilization of Telemedicine Virtual Visits in Pediatric Spinal Deformity Patients: A Comparison of Feasibility and Patient Satisfaction at a Large Academic Center. J Pediatr Orthop. (2020) 40(8):e712-e715. doi:10.1097/BPO.0000000000001553
100. Taddei M, Bulgheroni. Facing the real time challenges of the COVID-19 emergency for child neuropsychology service in Milan. Rs Dev Disabil. (2020) 107:1-6. doi:10.1016/j.ridd.2020.103786
101. Tan LO, Ganapathy S. A single centre study of the level of parents' satisfaction with the COVID-19 telemedicine consultation. Eur J Pediatr. (2023) 188(1):213-218. doi:10.1007/s00431-023-05276-7
102. Trace SL, Collinson A, Searle AJ, Lithander FE. Using videoconsultations to deliver dietary advice to children with chronic kidney disease: a qualitative study of parent and child perspectives. J Hum Nutr Diet. (2020) 33(6):881-889. Doi:10.1111/jhn.12750
103. Trivisano M, Specchio N, Pietrafusa N, Calabrese C, Ferretti A, Ricci R, Renzetti T, Raponi M, Vigevano F. Impact of COVID-19 pandemic on pediatric patients with epilepsy – The caregiver perspective. Epilepsy Behav. (2020) 113:1-5. doi:10.1016/j.yebeh.2020.107527
104. Tsai C, Savran A, Chau Y, Hurrell L, Forsyth C, Kumar H. Effectiveness of telehealth in providing preventive oral health care: A pilot evaluation of patient, carer, and clinician experience. J Public Health Dent. (2023) 83(4):331-339. doi:10.1111/jphd.12580
105. Tschamper MK Jakobsen R. Parents' experiences of videoconference as a tool for multidisciplinary information exchange for children with epilepsy and disability. J Clin Nurs. (2019) 28:1506-1516. doi:10.1111/jocn.14755
106. von Sengbusch S, Eisemann N, Mueller-Godeffroy E, Lange K, Doerdelmann J, Erdem A, Menrath I, Bokelmann J, Krasmann M, Kaczmarczyk P, Bertram B, Hiort O, Katalinic A, Frielitz FS. Outcomes of monthly video consultations as an add-on to regular care for children with type 1 diabetes: A 6-month quasi-randomized clinical trial followed by an extension phase. Pediatr Diabetes. (2020) 21(8):1502-1515. doi:10.1111/pedi.13133
107. von Sengbusch S, Doerdelmann J, Lemke S, Lange K, Hiort O, Katalinic A, Frielitz FS. Parental expectations before and after 12-month experience with video consultations combined with regular outpatient care for children with type 1 diabetes: a qualitative study. Diabet Med. (2021) 38(6): 1-12. doi:10.1111/dme.14410
108. von Sengbusch S, Schneidewind J, Bokelmann J, Scheffler N, Bertram B, Frielitz F, Hiort O, Lange K. Monthly video consultation for children and adolescents with Type 1 Diabetes mellitus during the COVID-10 pandemic. Diabetes Res Clin Pract. (2022) 193:1-6. doi:10.1016/j.diabres.2022.110135
109. Wade SL, Cassedy AE, Sklut M, Taylor G, McNally KA, Kirkwood MW, Stancin T, Kurowski BG. The relationship of adolescent and parent preferences for treatment modality with satisfaction, attrition, adherence, and efficacy: The coping with Head Injury through Problem-Solving (CHIPS) study. J Pediatr Psychol. (2019) 44(3):388-401. doi:10.1093/jpepsy/jsy087
110. Walijee H, Sood S, Markey A, Krishnan M, Lee A, De S. Is nurse-led telephone follow-up for post-operative obstructive sleep apnoea patients effective? A prospective observational study at a paediatric tertiary centre. Int J Pediatr Otorhinolaryngol. (2020) 129:1-5. doi:10.1016/j.ijporl.2019.109766
111. Weaver MS, Shostrom VK, Neumann ML, Robinson JE, Hinds PS. Homestead together: Pediatric palliative care telehealth support for rural children with cancer during home-based end-of-life care. Pediatr Blood Cancer. (2021) 68(4):1-9. doi:10.1089/jpm.2020.0617
112. Weaver MS, Jurgens A, Neumann ML, Schalley SM, Kellas JK, Navaneethan H, Tullis J. Actual Solidarity through Virtual Support: A Pilot Descriptive Study of an Online Support Group for Bereaved Parents. J Palliat Med. (2021) 24(8):1161-1165. doi:10.1089/jpm.2020.0617
113. Weber JC, Sohn K, Sauers-Ford HS, Hanhauser A, Tancredi DJ, Marcin JP, Hoffman KR. Impact of a Parent Video Viewing Program in the Neonatal Intensive Care Unit. Telemed J E Health. (2021) 27(6):679-685. doi:10.1089/tmj.2020.0251
114. Williams M. Using telehealth for rural paediatric diabetics: Does it deliver good care? J Paediatr Child Health. (2021) 57(1):109-113. doi:10.1111/jpc.15149
115. Yen S. Ranney ML, Krek M, Peters JR, Mereish E, Tezanos KM, Kahler CW, Solomon J, Beard C, Spirito A. Skills to enhance positivity in suicidal adolescents: Results from a pilot randomized clinical trial. J Posit Psychol. (2020) 15(3):348-361. doi:10.1080/17439760.2019.1615105
116. Young K, Gupta A, Palacios R. Impact of Telemedicine in Pediatric Postoperative Care. Telemed J E Health. (2019) 25(11):1083-1089. doi:10.1089/tmj.2018.0246
117. Zayde A, Kilbride A, Kucer A, Willis HA, Nikititiades A, Alpert J, Gabbay V. Connection During COVID-19: Pilot Study of a Telehealth Group Parenting Intervention. Am J Psychotherap. (2022) 75(2):67-74. Doi:10.1176/appi.psychotherapy.20210005
