# Supplementary material for: Synchronous virtual care in children’s health care: a scoping review
Source: Front Pediatr. 2025 Nov 6;13:1610407. doi: 10.3389/fped.2025.1610407 (PMC12631453; doi:10.3389/fped.2025.1610407)
Supplement: Supplementary file 2 [file Table2.docx]

| **Section of the Paper** | **Study Characteristic** | **List of Studies Mentioning this Characteristic** |
| --- | --- | --- |
| 3.1. How has virtual care been implemented? | Virtual care implemented through video-conferencing as the mode of communication | 1-80 |
|  | Virtual care implemented through a range of approaches whereby patients and families could choose | 20-28, 59, 67, 81-83 |
|  | Virtual care implemented where parents/caregivers were required to gather and upload child/patient information to an online portal or send it via email pre/post appointment | 16, 23, 44, 60, 63, 64, 69, 72, 78, 83-89 |
|  | Virtual care implemented whereby modalities are contrasted (e.g., in-person vs. virtual) | 20-28, 73, 81-83, 93 |
| 3.2. What are study objectives and the aims of the virtual care applications? | Explored experiences of, or level of satisfaction with, virtual care | 16, 17, 20, 22, 29, 30, 50, 51, 53, 54, 60, 62, 65, 71-73, 75-77, 79, 80, 83, 86-89, 94-103 |
|  | Evaluated satisfaction with virtual care | 1, 3, 4, 6, 7, 9-12, 15, 23-26, 31-38, 52, 59, 66, 67, 69, 70, 104-109 |
|  | Compared virtual care with face-to-face care approaches | 9, 11, 12, 20, 24, 27, 28, 31, 34, 45, 47, 57, 73, 81, 83, 90-93 |
|  | Developed virtual care intervention to continue care and/or offer care during the COVID-19 pandemic | 10, 11, 13, 16, 19, 28, 39-41, 59, 66, 67, 69, 90, 105-109 |
| 3.3. By whom, when, where and how has virtual care been evaluated? | Virtual care experiences were largely gathered from family caregivers solely | 2-4, 7, 9, 11, 17, 20-23, 25, 29, 35, 38, 39, 45, 46, 49, 54, 58, 59, 64-66, 72, 75-77, 80, 83, 87, 88, 90, 91, 93. 98, 99, 102-104, 111, 112 |
|  | Virtual care experiences were gathered from both family caregivers and children/youth | 1, 5, 6, 10, 14-16, 18, 19, 24, 27, 30-34, 36, 37, 42, 44, 47, 48, 50-52, 57, 60-62, 67-71, 73, 74, 78, 79, 86, 89, 92, 94, 97, 100, 101, 106, 107, 109, 114-116 |
|  | Virtual care experiences were gathered from children/youth solely | 8, 12, 13, 26, 28, 43, 53, 55, 56, 61, 70, 74, 84, 100, 115 |
|  | Articles published in 2013 | 23, 47 |
|  | Articles published in 2015 | 82, 104, 111 |
|  | Articles published in 2016 | 44 |
|  | Articles published in 2017 | 99, 110 |
|  | Articles published in 2018 | 5, 50, 56 |
|  | Articles published in 2019 | 53, 55, 61, 64, 112 |
|  | Articles published in 2020 | 1, 24, 27, 30, 35, 37, 40, 45, 46, 48, 52, 62, 78, 87, 94, 97, 102, 105, 111, 114, 116 |
|  | Articles published in 2021 | 2, 6, 8, 13, 17, 18, 21, 26, 33, 34, 41, 42, 54, 58, 59, 60, 67, 69, 79, 88, 92, 98, 101, 104, 115 |
|  | Articles published in 2022 | 3, 4, 9-11, 14, 16, 19, 20, 23-25, 29, 31 |
|  | Articles published in 2023 | 3, 4, 9-11, 14, 16, 19, 20, 22-25, 29, 31, 32, 39, 51, 57, 63, 68, 70, 76, 81, 86, 93, 100, 103, 107, 108 |
|  | Used a quantitative approach that drew on data collected during the COVID-19 pandemic | 1-43 |
|  | Articles were based in the United States | 3, 5, 8-10, 12, 17-19, 23-25, 31, 32, 42, 45, 49-51, 53-55, 57, 61, 68, 69, 73, 80, 85, 89, 90, 94, 96, 97, 99, 101, 104, 109, 111,112 |
|  | Used a quantitative approach with an observational design | 1-3, 6-9, 12, 15, 16, 21, 22, 24, 26-28, 30-34, 36, 38-40, 44-48, 50, 59, 81, 94, 104, 113, 114 |
|  | Used a quantitative approach with an exploratory design | 5, 10, 13, 16, 17, 18, 20, 23, 29, 37, 51-55, 82, 95, 96, 110, 111, 116 |
|  | Used a quantitative approach with an experimental design | 4, 17, 19, 56-58, 97, 115 |
|  | Used surveys to collect data | 1-16, 19, 21-25, 27, 29-38, 40-42, 45-47, 49-52, 54-58, 81, 84, 94, 95, 97, 104, 105, 110, 112, 116 |
|  | Used observation data | 13, 28, 48, 114 |
|  | Used mixed methods | 16, 60, 61, 66, 67, 69, 70, 71, 73, 76, 83, 85, 88, 90, 92, 99-103, 108 |
|  | Used a range of qualitative approaches | 59, 62-65, 68, 86, 87, 98, 105-107, 112, 115, 117 |
| 3.4. How does virtual care address the social determinants of health? | Based at urban hospitals | 2, 6-12, 14, 15, 17, 20, 21, 23, 28, 32-35, 38, 47, 53, 54, 56, 61, 64, 67-69, 71, 82, 83, 87, 91, 94-98, 101, 103, 104, 108, 113 |
|  | Required participants to read and speak a specific language | 6, 17, 19, 29, 53, 56, 60, 64, 78, 83, 84, 86, 88, 91, 97, 99, 110, 111 |
|  | Participants/families were variably required to have an electronic/technology device | 4, 43, 54, 56, 66, 68, 84 |
|  | Participants were rendered ineligible from study participation if lacking technology/technology access | 22, 40, 47, 55, 60, 61, 69, 78, 83, 88, 106 |
| 3.5. How do families experience virtual care? | Reported benefits of virtual care interventions | 1-10, 12- 19, 21-23, 25-28, 30-33, 35-38, 40, 41, 43-52, 54-58, 61-63, 65-74, 80-86, 88-90, 92, 94-102, 104-111, 113-116 |
|  | Reported challenges of virtual care interventions | 1, 2, 6-8, 10, 13, 15, 16, 19-23, 28, 30, 31, 33, 38, 40-43, 45-48, 50-52, 54-56, 58, 61-63, 65-74, 80, 81, 83, 85, 86, 88, 89, 90, 92, 95, 98, 99, 100-102, 104-110, 114-117 |
|  | Reported satisfaction for virtual care interventions | 1, 2, 4-7, 10-12, 14-16, 20, 21, 23, 25, 26, 31-41, 44-46, 49, 52, 57, 62-64, 66, 68, 69, 70, 71, 73, 74, 76, 78, 83, 91, 92, 95, 96, 100-105, 107-110, 113, 116 |
|  | Reported utilization rates | 3, 6, 24, 27, 29-31, 34, 37, 40-42, 45, 51, 55, 61, 67, 69, 72, 73, 82, 83, 85, 88, 90, 91, 93, 95, 99, 100, 102, 104, 109 |
|  | Reported on approach preferences | 7, 23, 27, 29, 34, 39, 40, 43, 44, 46, 47, 61-63, 69, 71, 73, 83, 88, 89, 91, 92, 94, 100, 103,104, 105, 108, 109,110, 111, 112, 115 |
| 3.5. How do families experience virtual care |  |  |
| 3.5.1. Benefits of virtual care | Reported one or more benefits of virtual care | 1-10, 12-19, 21-23, 25-28, 30-33, 35-41, 43-50, 51, 52, 54-58, 61-63, 65-72, 74, 80-86, 88-90, 92, 94-102, 104-111, 113-116 |
| 3.5.2. Satisfaction with virtual care | Focused on virtual care satisfaction | 1, 2, 4-7, 10-12, 14-16, 20, 21, 23, 25, 26, 31-41, 44-46, 49, 51, 52, 57, 62-64, 66, 68-71, 73, 74, 76, 78, 83, 91, 92, 95, 96, 100-105, 107-110, 114,116 |
|  | 82.1% of quantitative studies indicated satisfaction with the care received | 2, 5-7, 10-12, 15, 16, 20, 21, 25, 26, 31-33, 36-41, 44-46, 52, 57, 95, 96, 104, 114, 116 |
|  | 87.2% of quantitative studies indicated satisfaction with the platform/approach used | 1, 2, 4-7, 11, 12, 14-16, 20, 21, 23, 25, 26, 31, 33, 36-39, 41, 44-46, 49, 57, 95, 96, 104,116 |
| 3.5.3. Challenges with virtual care: families | Reported challenges largely attributed to difficulties with technology | 1, 2, 6-8, 10, 13, 15, 16, 19-23, 28, 30, 31, 33, 38, 40-43, 45-48, 50, 51, 52, 54-56, 58, 61-63, 65-74, 80, 81, 83, 85-90, 95, 98-102, 104-110, 113, 115-117 |
|  | Quantitative articles reported some degree of technology barriers/issues | 1, 7, 10, 13, 14, 16, 19, 21-23, 28, 30, 38, 42, 47-52, 54, 55, 58, 81, 95, 110, 115, 116 |
| 3.5.6. What advice do families offer in delivering virtual care? | Identified parent/caregiver and child preferences for virtual care design and implementation | 7, 23, 27, 29, 34, 39, 40, 43, 44, 46, 47, 50, 61-63, 69, 71, 73, 83, 88, 89, 91, 92, 94, 100, 103-105, 108-112, 115 |

Reference List

1. Assenza, C, Catania H, Antenore C, Gobbetti T, Gentili P, Paolucci S, Morelli D. Continuity of Care During COVID-19 Lockdown: A Survey on Stakeholders' Experience with Telerehabilitation. Front Neurol. (2021) 11:1-10. doi:10.3389/fneu.2020.617276
2. Bate NJ, Xu SC, Pacilli M, Roberts LJ, Kimber C, Nataraja RM. (2021). Effect of the COVID-19 induced phase of massive telehealth uptake on end-user satisfaction. Intern Med J. (2021) 51(2):206-214. doi:10.1111/imj.15222
3. Cockrell H, Wayne D, Wandell G, Wang X, Greenberg SLM, Kieran K, Dick A, Bonilla-Velez J. Understanding hispanic patient satisfaction with telehealth during COVID-19. J Pediatr Surg. (2022) 58(9):1783-1788. doi:10.1016/j.pedsurg.2022.12.006
4. Giuseppe DB, Giuseppina N, Desiree S, Angela S, Maurizio G, Perrone S. Improving Care in Neonatal Intensive Units During the COVID-19 Pandemic: A Survey on Electronic Health Communication. J Intensive Care Med. (2022) 37(5):671-678. doi:10.1177/08850666211055058
5. Guttmann-Bauman I, Kono J, Lin AL, Ramsey KL, Boston BA. Use of Telehealth Videoconferencing in Pediatric Type 1 Diabetes in Oregon. Telemed J E Health. (2018) 24(1):86-88. doi:10.1089/tmj.2017.0072
6. Holzman SA, Davis-Dao CA, Khoury AE, Fortier MA, Kain, NZ. Telemedicine and patient satisfaction during the COVID-19 pandemic: A case-control study of outpatient pediatric urology patients. J Child Health Care. (2021) 27(3):351-359. Doi:10.1177/13674935211058272
7. Kilipiris EG, Horn F, Kolnikova M, Ochoa JV, Matuskova O, Jelovac D, Stebel A. Parental satisfaction from telemedicine in the follow-up of children operated for craniosynostosis during COVID-19 pandemic. Cleft Palate J. (2023) 60(5):562-568. doi:10.1177/10556656221074214
8. Kramer JL, De Asis K. Osteopathic interventions via telehealth in a pediatric population: a retrospective case series. J Osteopath Med. (2021) 121(11):857-861. Doi:10.151/jom-2021-0124
9. McCoy J, Shaffer J, Amber D, Dohar JE. Pediatric otolaryngology telemedicine amid a pandemic - And beyond. Int J Pediatr Otorhinolaryngol. (2022) 153:1-7. doi:10.1049/j.ijporl.2021.111014
10. McNally Keehn R, Enneking B, James C, Tang Q, Rouse M, Hines E, Raches C, Etling A. Telehealth evaluation of pediatric neurodevelopmental disabilities during the COVID-19 pandemic: Clinician and caregiver perspectives. J Dev Behav Pediatr. (2022) 43(5):262-272. doi:10.1097.DBP.0000000000001043
11. Meininger L, Adam J, von Wirth E, Viefhaus P, Woitecki K, Walter D, Döpfner M. Cognitive-behavioral teletherapy for children and adolescents with mental disorders and their families during the COVID-19 pandemic: a survey on acceptance and satisfaction. Child Adolesc Psychiatry Ment Health. (2022) 16(1):1-11. Doi:10.1189/s13034-022-00494-7
12. Netson RA, Miller S, Incorvia J, Shah A, Estrada CR, Toomey SL, Taghinia AH. Patient experience with virtual preoperative consultations in pediatric surgical specialties. J Pediatr Surg. (2023) 58(9):1776-1782. doi:10.1016/j.jpedsurg.2022.12.027
13. Onofri A, Pavone M, De Santis S, Verrillo E, Caggiano S, Ullmann N, Cutrera R. Telemedicine in children with medical complexity on home ventilation during the COVID‐19 pandemic, Pediatr Pulmonol. (2021) 56(6):1395-1400. doi: 10.1002/ppul.25289
14. Severini RDSG, Oliveira PCD, Couto TB, Simon Junior H, Andrade APMD, Nanbu DY, Farhat SCL, Schvartsman C. Fast, cheap and feasible: Implementation of pediatric telemedicine in a public hospital during the Covid-19 pandemic. J Pediatr (Rio J). (2022) 98(2):183-189. doi:10.1016/j.jped.2021.05.007
15. Tan LO, Ganapathy S. A single centre study of the level of parents' satisfaction with the COVID-19 telemedicine consultation. Eur J Pediatr. (2023) 188(1):213-218. doi:10.1007/s00431-023-05276-7
16. von Sengbusch S, Schneidewind J, Bokelmann J, Scheffler N, Bertram B, Frielitz F, Hiort O, Lange K. Monthly video consultation for children and adolescents with Type 1 Diabetes mellitus during the COVID-10 pandemic. Diabetes Res Clin Pract. (2022) 193:1-6. doi:10.1016/j.diabres.2022.110135
17. Weaver MS, Jurgens A, Neumann ML, Schalley SM, Kellas JK, Navaneethan H, Tullis J. Actual Solidarity through Virtual Support: A Pilot Descriptive Study of an Online Support Group for Bereaved Parents. J Palliat Med. (2021) 24(8):1161-1165. doi:10.1089/jpm.2020.0617
18. Weaver MS, Shostrom VK, Neumann ML, Robinson JE, Hinds PS. Homestead together: Pediatric palliative care telehealth support for rural children with cancer during home-based end-of-life care. Pediatr Blood Cancer. (2021) 68(4):1-9. doi:10.1089/jpm.2020.0617
19. Zayde A, Kilbride A, Kucer A, Willis HA, Nikititiades A, Alpert J, Gabbay V. Connection During COVID-19: Pilot Study of a Telehealth Group Parenting Intervention. Am J Psychotherap. (2022) 75(2):67-74. Doi:10.1176/appi.psychotherapy.20210005
20. Hallford HG, Szyld E, McCoy M, Makkar A. A 360 Evaluation of Neonatal Care Quality at a Level II Neonatal Intensive Care Unit when Delivered Using a Hybrid Telemedicine Service. Am J Perinatol. (2022) Nov 10:1-8. doi:10.1055/a-1932-9921
21. Hendra K, Neemuchwala F, Chan M, Ly NP, Gibb ER. Patient and Provider Experience With Cystic Fibrosis Telemedicine Clinic. Front Pediatr. (2021) 9:1-7. doi:10.3389/fped.2021.784692
22. Hiscock H, Pelly R, Hua X, West S, Tucker D, Raymundo CM, Dalziel K. Survey of paediatric telehealth benefits from the caregiver perspective. Aust Health Rev. (2022) 46(2):197-203. doi: 10.1071/AH21036
23. Love M, Hunter AK, Lam G, Muir LV, Lin HC. Patient satisfaction and perceived quality of care with telemedicine in a pediatric gastroenterology clinic. Pediatr Rep. (2022) 14(2):181-189. doi:10.3390/pediatric14020025
24. Marques S, Cruz JAW, da Cunha MAVC, Tuon FF, de Moraes TP, Zdziarski AD, Bomher ST, Donnelly LF, Capasso R. Patient and family experience with telemedicine and in-person pediatric and obstetric ambulatory encounters throughout 2020, during the COVID-19 epidemic: the distance effect. BMC Health Serv Res. (2022) 22(1):1-8. doi:10.1186/s12913-022-08037-8
25. Olateju A, Cervantes M, Dowshen N. Kuhns LM, Dhar CP. Acceptability of Telemedicine Among Parents of Adolescent Patients in an Adolescent Clinic: Cross-sectional Survey Study. JMIR Pediatr Parent. (2022) 5(4):1-7. doi:10.2196/39704
26. Reid S, Bhatt M, Zemek R, Tse S. Virtual care in the pediatric emergency department: a new way of doing business? CJEM. (2021) 23(1):80-84. doi:10.1007/s43678-020-00048-w
27. Sharma S, Daniel M. Telemedicine in paediatric otorhinolaryngology: Lessons learnt from remote encounters during the Covid19 pandemic and implications for future practice. Int J Pediatr Otorhinolaryngol. (2020) 139:1-6. doi:10.1016/j.ijporl.2020.110411
28. Shulman J, Conroy C, Bento S, Bryant G, Jervis K, Sethna NF. Pediatric pain rehabilitation during the COVID-19 pandemic: exploring the effectiveness of a hybrid intensive interdisciplinary pain treatment model. Disabil Rehabil. (2023) 45(19):3079-3086. doi:10.1080/09638288.2022.2125083
29. Lawrence J, Measey MA, Hoq M, Hiscock H, Rhodes A. Virtual health care for children: Parental willingness to adopt virtual health-care technologies. J Paediatr Child Health. (2022) 58(8):1323-1329. doi:10.1111/jpc.15974
30. Singh N, Datta M. Single-centre telephone survey on patients' perspectives regarding remote paediatric outpatient consultations in a district general hospital. BMJ Paediatr. (2020) 4(1):1-2. doi:10.1136/bmjpo-2020-000885
31. Hoi KK, Brazina SA, Kolar-Anderson R, Zopf DA, Bohm LA. A Longitudinal Comparison of Telemedicine Versus In-Person Otolaryngology Clinic Efficiency and Patient Satisfaction During COVID-19. Ann Otol Rhinol Laryngol. (2022) 131(11):1177-1184. doi:10.1177/00034894211055349
32. Jones E, Kurman J, Delia E, Crockett J, Peterson R, Thames J, Salorio C, Kalb L, Jacobson L, Stone J, Zabel TA. Parent satisfaction with outpatient telemedicine services during the COVID-19 pandemic: A repeated cross-sectional study. Front Pediatr. (2022) 10:1-9. doi:10.3389/fped.2022.908337
33. Lakshin G, Banek S, Keese D, Rolle U, Schmedding A. Telemedicine in the pediatric surgery in Germany during the COVID-19 pandemic. Pediatr Surg Int. (2021) 37(3):389-395. doi:10.1007/s00383-020-04822-w
34. Lo WB, Herbert K, Rodrigues D. Clinical effectiveness of and family experience with telephone consultation in a regional pediatric neurosurgery center in the United Kingdom, Journal of neurosurgery. Pediatr. (2021) 28(4):483-489. doi:10.3171/2021.3.PEDS20862
35. Odeh R, Gharaibeh L, Daher A, Kussad S, Alassaf A. Caring for a child with type 1 diabetes during COVID-19 lockdown in a developing country: Challenges and parents' perspectives on the use of telemedicine. Diabetes Res Clin Pract. (2020) 168:1-7. doi:10.1016/j.diabres.2020.108393
36. Sikka K. Parent's perspective on teletherapy of pediatric population with speech and language disorder during Covid-19 lockdown in India. Indian J Otolaryngol Head Neck Surg. (2023) 75(1):14-20. doi:10.1007/s12070-022-03310-y
37. Trivisano M, Specchio N, Pietrafusa N, Calabrese C, Ferretti A, Ricci R, Renzetti T, Raponi M, Vigevano F. Impact of COVID-19 pandemic on pediatric patients with epilepsy – The caregiver perspective. Epilepsy Behav. (2020) 113:1-5. doi:10.1016/j.yebeh.2020.107527
38. Alghamdi SA. Parent perceptions regarding virtual pediatric dental clinics during COVID-19 pandemic: a cross-sectional study. Peer J. (2023) 11:1-14. Doi:10.7717/peerj.15289
39. Alzahrani AM, Magliah SF, Turkistani HA, Abulaban BA, Sabban MF, Mashat MA, Al Shaikh AM. Perception of primary caregiver toward virtual pediatric clinics for type 1 diabetes mellitus during COVID-19 pandemic in Jeddah, Saudi Arabia: A cross-sectional study. Ann Med Surg. (2022) 81:1-6. Doi:10.1016/j.amsu.2022.104550
40. Darr A, Senior A, Argyriou K, Limbrick J, Nie H, Kantczak A, Stephenson K, Parmar A, Grainger J. The impact of the coronavirus (COVID-19) pandemic on elective paediatric otolaryngology outpatient services – An analysis of virtual outpatient clinics in a tertiary referral centre using the modified paediatric otolaryngology telemedicine satisfaction survey (POTSS). Int J Pediatr Otorhinolaryngol. (2017) 138:1-8. doi:10.1016/j.ijporl.2020.110383
41. Davis J, Gordon R, Hammond A, Perkins R, Flanagan F, Rabinowitz E, Simoneau T, Sawicki GS. Rapid Implementation of Telehealth Services in a Pediatric Pulmonary Clinic During COVID-19. Pediatr. (2021) 148(1):1-9. doi:10.1542/peds.2020-030494
42. Kolb CM, Born K, Banker K, Barth PC, Aaronson NL. Improving attendance and patient experiences during the expansion of a telehealth-based pediatric otolaryngology practice. Otolaryngol Head Neck Surg. (2021) 164(5):952-958. doi:10.1177/0194599820965917
43. Bales NJ, Perera DC, Foerster R, Poirier L, Ducis K. (2023). Analysis of a novel virtual pediatric concussion clinic in a rural setting. Childs Nerv Syst*.* (2023) 1-7. doi:10.1007/s00381-023-06231-5
44. Bianciardi Valassina MF, Bella S, Murgia F, Carestia A, Prosseda E. Telemedicine in pediatric wound care. Clin Ter. 2016;167(1 Supplement), 21-23. doi: 10.7417/T.2016.1915Bian
45. Finkelstein JB, Cahill D, Young K, Humphrey K, Campbell CS, Nelson CP, Gupta A, Estrada CR. Telemedicine for Pediatric Urological Postoperative Care is Safe, Convenient and Economical. J Urol. (2020) 204(1):144-148. doi:10.1097/JU.0000000000000750
46. Fortini S, Espeche A, Caraballo R. Telemedicine and epilepsy: A patient satisfaction survey of a pediatric remote care program. Epilepsy Res. (2020) 165:1-5. doi:10.1016/j.eplepsyres.2020.106370
47. Jury SC, Walker AM, Kornberg AJ. The introduction of web-based video-consultation in a paediatric acute care setting. J Telemed Telecare. (2013) 19(7):383-387. Doi:10.1177/1357633X13506530
48. Khoury M, Phillips DB, Wood PW, Mott WR, Stickland MK, Boulanger P, Rempel GR, Conway J, Mackie, AS, Khoo NS. Cardiac rehabilitation in the paediatric Fontan population: development of a home-based high-intensity interval training programme. Cardiol Young. (2020) 30:1409-1416. doi:10.1017/sS1047951120002097
49. Makkar A, McCoy M, Hallford G, Foulks A, Anderson M, Milam J, Wehrer M, Doerfler E, Szyld E. Evaluation of neonatal services provided in a level II NICU utilizing hybrid telemedicine: A prospective study. Telemed J E Health. 2020;26(2), 176-183. doi:10.1089/tmj.2018.0262
50. Qubty W, Patniyot I, Gelfand A. Telemedicine in a pediatric headache clinic. Neurology. (2018) 90(19):E1702-E1705. doi:10.1212/WNL.0000000000005482
51. Stewart C, Coffey-Standoval J, Souverein EA, Ho TC, Lee TC, Nallasamy S. Patient and Provider Experience in Real-Time Telemedicine Consultations for Pediatric Ophthalmology. Clin Ophthalmol. (2022) 16:2943-2953. doi:10.2147/OPTH.S374811
52. Taddei M, Bulgheroni. Facing the real time challenges of the COVID-19 emergency for child neuropsychology service in Milan. Rs Dev Disabil. (2020) 107:1-6. doi:10.1016/j.ridd.2020.103786
53. Wade SL, Cassedy AE, Sklut M, Taylor G, McNally KA, Kirkwood MW, Stancin T, Kurowski BG. The relationship of adolescent and parent preferences for treatment modality with satisfaction, attrition, adherence, and efficacy: The coping with Head Injury through Problem-Solving (CHIPS) study. J Pediatr Psychol. (2019) 44(3):388-401. doi:10.1093/jpepsy/jsy087
54. Weber JC, Sohn K, Sauers-Ford HS, Hanhauser A, Tancredi DJ, Marcin JP, Hoffman KR. Impact of a Parent Video Viewing Program in the Neonatal Intensive Care Unit. Telemed J E Health. (2021) 27(6):679-685. doi:10.1089/tmj.2020.0251
55. Young K, Gupta A, Palacios R. Impact of Telemedicine in Pediatric Postoperative Care. Telemed J E Health. (2019) 25(11):1083-1089. doi:10.1089/tmj.2018.0246
56. Chalmers JA, Sansom-Daly UM, Patterson P, McCowage G, Anazodo A. Psychosocial assessment using telehealth in adolescents and young adults with cancer: A partially randomized patient preference pilot study. J Med Internet Res. (2018) 7(8):1-25. doi:10.2196/resprot.8886
57. Elbin RJ, Stephenson K, Lipinski D, Maxey K, Womble MN, Reynolds E, Covert K, Kontos AP. In-Person Versus Telehealth for Concussion Clinical Care in Adolescents: A Pilot Study of Therapeutic Alliance and Patient Satisfaction. J Head Trauma Rehab. (2022) 37(4):213-219. doi:10.1097/HTR.0000000000000707
58. Rashid M, Haskett J, Parkinson McGraw L, Noble A, van Limbergen J, Otley A. Teaching Families of Children with Celiac Disease about Gluten-Free Diet Using Distributed Education: a Pilot Study. Can J Diet Pract Res. (2021) 82(1):38-40. doi:10.3148/cjdpr-2020-021
59. Lanzarin CMV, von Wangenheim A, Rejane-Heim TC, Nascimento FS, Wagner HM, Abel HS, Junior JDDS, Xikota JC. Teleconsultations at a Pediatrics Outpatient Service in COVID-19 Pandemic: First Results. Telemed J E Health. (2021) 27(11):1311-1316. doi:10.1089/tmj.2020.0471
60. von Sengbusch S, Doerdelmann J, Lemke S, Lange K, Hiort O, Katalinic A, Frielitz FS. Parental expectations before and after 12-month experience with video consultations combined with regular outpatient care for children with type 1 diabetes: a qualitative study. Diabet Med. (2021) 38(6): 1-12. doi:10.1111/dme.14410
61. Kelly SL, Steinberg EA, Suplee A, Upshaw NC, Campbell KR, Thomas JF, Buchanan CL. Implementing a Home-Based Telehealth Group Adherence Intervention with Adolescent Transplant Recipients. Telemed J E Health. (2019) 25(11):1040-1048. doi:10.1089/tmj.2018.0164
62. Trace SL, Collinson A, Searle AJ, Lithander FE. Using video consultations to deliver dietary advice to children with chronic kidney disease: a qualitative study of parent and child perspectives. J Hum Nutr Diet. (2020) 33(6):881-889. Doi:10.1111/jhn.12750
63. Doerdelman J, Frielitz FS, Lange K, Meinsen T, Reimers S, Ottersberg T, Katalinic A, Hiort O, Von Sengbusch S. Video Consultation for Parents with a Child Newly Diagnosed with Type 1 Diabetes: A Qualitative Study. Exp Clin Endocrinol Diabetes. (2022) 130(8):519-524. doi:10.1055/a-1655-5471
64. Garne Holm, K, Brodsgaard A, Zachariassen G, Smith AC, Clemensen J. Parent perspectives of neonatal tele-homecare: A qualitative study. J Telemed Telecare. (2019) 25(4):221-229. doi:10.1177/1357633X18765059
65. Tschamper MK Jakobsen R. Parents' experiences of videoconference as a tool for multidisciplinary information exchange for children with epilepsy and disability. J Clin Nurs. (2019) 28:1506-1516. doi:10.1111/jocn.14755
66. Blagdon A, Smith D, Bramfield T, Soraisham A, Mehrem AA. Evaluation of family and staff experiences with virtual rounding and bedside presence in a tertiary neonatal intensive care unit during the COVID-19 pandemic. J Telemed Telecare. (2022) 1-15. doi:10.1177/1357633X221081294
67. Gefen N, Steinhart S, Beeri M, Weiss PL. Lessons learned during a naturalistic study of online treatment for pediatric rehabilitation. Int J Environ Res Public Health. (2021) 18:1-17. doi:10.3390/ijerph18126659
68. Hoyt-Austin AE, Miller IT, Kuhn-Riordon KM, Rosenthal JL, Chantry CJ, Marcin JP, Hoffman KR, Kair LR. Bonding, Relaxation, Separation, and Connection: Expressing Human Milk While Videoconferencing with the Hospitalized Premature Infant. Breastfeed Med. (2022) 17(8):653-659. doi:10.1089/bfm.2021.0214
69. Gan Z, Lee SY, Weiss DA, Van Batavia J, Siu S, Frazier J, Zderic SA, Shukla AR, Srinivasan AK, Kolon TF, Zaontz MR, Canning DA, Long CJ. Single institution experience with telemedicine for pediatric urology outpatient visits: Adapting to COVID-19 restrictions, patient satisfaction, and future utilization. J Pediatr Urol. (2021) 17(4):480.e1-480.e7. doi:10.1016/j.jpurol.2021.05.012
70. Huscsava MM, Scharinger C, Plener PL, Kothgassner OD. The world somehow stopped moving: Impact of the COVID-19 pandemic on adolescent psychiatric outpatients and the implementation of teletherapy. Child and Adolesc Ment Health. (2022) 27(3):232-237. doi:10.1111.camh.12481
71. Ruskin D, Tremblay M, Szczech K, Rosenbloom BN, Mesaroli G, Sun N, D'Alessandro L. Virtual multidisciplinary pain treatment: Experiences and feedback from children with chronic pain and their caregivers. Physiother Theory Pract. (2023) 1-22. doi:10.1080/09593985.2023.2171750
72. Tsai C, Savran A, Chau Y, Hurrell L, Forsyth C, Kumar H. Effectiveness of telehealth in providing preventive oral health care: A pilot evaluation of patient, carer, and clinician experience. J Public Health Dent. (2023) 83(4):331-339. doi:10.1111/jphd.12580
73. Sinha N, Cornell M, Wheatley B, Munley N, Seeley M. Looking Through a Different Lens: Patient Satisfaction With Telemedicine in Delivering Pediatric Fracture Care. J Am Acad Orthop Surg Glob Res Rev. (2019) 3(9):1-6. doi: 10.5435/JAAOSGlobal-D-19-00100
74. Costa-Cordella S, Carmona KB, Contreras ND, Cano AG, Bonta CM, Grasso-Cladera A. Digital adaptation of group activities in an outpatient center of a children’s hospital: Adolescent participants’ experiences. Medwave. (2023) 23(4):1-10. doi:10.5867/medwave/2023.01.25686
75. Mateus LA, Law MP, Khowaja AR, Orlando E, Pace A, Roy M, Sulowski C. Examining perceptions of a telemedicine network for pediatric emergency medicine: A mixed-methods pilot study. Front Digit Health. (2023) 5:1-8. doi:10.3389/fdgth.2023.1181059
76. Mena R, Mendoza E, Mangano FT, Henrickson M, Scott M, DeFoor WR, Little KJ, Ruschman J, Prada CE. International Pediatric Multidisciplinary Management Using Telemedicine to Promote Equitable Care. Telemed J E Health. (2022) 29(5):674-685. doi:10.1089/tmj.2022.0165
77. Shamsi A, Namnabati M, Ehteshami A, Zandi H. Telehealth experiences of mothers of hospitalized and discharged preterm infants in Islamic Republic of Iran. East Mediterr Health J. (2023) 29(5):309-316. doi:10.26719/emhj.23.055
78. von Sengbusch S, Eisemann N, Mueller-Godeffroy E, Lange K, Doerdelmann J, Erdem A, Menrath I, Bokelmann J, Krasmann M, Kaczmarczyk P, Bertram B, Hiort O, Katalinic A, Frielitz FS. Outcomes of monthly video consultations as an add-on to regular care for children with type 1 diabetes: A 6-month quasi-randomized clinical trial followed by an extension phase. Pediatr Diabetes. (2020) 21(8):1502-1515. doi:10.1111/pedi.13133
79. Williams M. Using telehealth for rural paediatric diabetics: Does it deliver good care? J Paediatr Child Health. (2021) 57(1):109-113. doi:10.1111/jpc.15149
80. Smith SL, Vaquerano J, Humphreys BP, Aytur SA. Parent satisfaction with a telehealth parent coaching intervention to support family participation. OTJR. (2023) 43(3):531-539. doi:10.1177/15394492231164942
81. Frye WS, Gardner L, Mateus JS. Utilising telemental health in a paediatric outpatient psychology clinic: Therapeutic alliance and outcomes. Couns Psychother Res. (2022) 22(2):322-330. doi:10.1002/capr.12450
82. Akobeng AK, O’Leary N, Vail A, Brown N, Widiatmoko D, Fagbemi A, Thomas AG. Telephone Consultation as a Substitute for Routine Out-patient Face-to-face Consultation for Children With Inflammatory Bowel Disease: Randomised Controlled Trial and Economic Evaluation. EbioMedicine. (2015) 2(9):1251-1256. doi:10.1016/j.ebiom.2015.08.011
83. Gund A, Sjöqvist BA, Wigert H, Hentz E. A randomized controlled study about the use of eHealth in the home health care of premature infants. BMC Med Inform Decis Mak. (2013) 13(1):1-11. doi:10.1186/1472-6947-13-22
84. Nguyen HH, Van Hare GF, Rudokas M, Bowman T, Silva JNA. SPEAR trial: Smartphone pediatric electrocardiogram trial. PloS One. (2015) 10(8):1-9. Doi:10.1371/journal.pone.0136256
85. Stagg A, Giglia TM, Gardner MM, Offit BF, Fuller KM, Natarajan SS, Hehir DA, Szwast AL, Rome JJ, Ravishankar C, Laskin BL, Preminger TJ. Initial Experience with Telemedicine for Interstage Monitoring in Infants with Palliated Congenital Heart Disease. Pediatr Cardiol. (2023) 44(1):196-203. doi:10.1007/s00246-022-02993-y
86. Hylén M, Nilsson S, Kristensson-Hallstrὂm I, Kristjánsdóttir G, Stenstrὂm Vilhjálmsson R. Access to health care perceived by parents caring for their child at home supported by eHealth – a directed approach introducing aperture. BMC Health Serv Res. (2022) 22:1-11. doi:10.1186/s12813-022-08398-0
87. Strand AS, Johnsson B, Hena M, Magnusson B, Hallstrom I.K. Developing eHealth in neonatal care to enhance parents' self-management. Scan J Caring Sci. (2022) 36(4):969-977. doi:10.1111/scs.12994
88. Lindkvist RM, Sjostrom-Strand A, Landgren K, Johnsson BA, Stenstrom P, Hallstrom IK. "In a Way We Took the Hospital Home"-A Descriptive Mixed-Methods Study of Parents' Usage and Experiences of eHealth for Self-Management after Hospital Discharge Due to Pediatric Surgery or Preterm Birth. Int J Environ res Public Health. (2021) 18:1-13. doi:10.3390/ijerph18126480
89. Caruso CG, Warren JB, Carney PA. Parent experiences of a remote patient monitoring program enabling early discharge from the neonatal intensive care unit with nasogastric tube feeding. J Neonatal Perinatal Med. (2023) 16:301-309. doi:10.3233/NPM-221181
90. Sprecher E, Conroy K, Krupa J, Shah S, Chi GW, Graham D, Starmer AJ. A Mixed-Methods Assessment of Coronavirus Disease of 2019-Era Telehealth Acute Care Visits in the Medical Home. J Pediatr. (2023) 255:121-127. doi:10.1016/j.jpeds.2022.10.036
91. Lee S, Dick BD, Jordan A, McMurty CM. A Parent-Targeted Group Intervention for Pediatric Pain Delivered In-Person or Virtually: Feasibility, Acceptability, and Effectiveness. Clin J Pain. (2023) 39(3):127-137. doi:10.1097/AJP.0000000000001100
92. Brothwood PL, Baudinet J, Stewart CS, Simic M. Moving online: young people and parents' experiences of adolescent eating disorder day programme treatment during the COVID-19 pandemic. J Eat Disord. (2021) 9(1):1-10. doi:10.1186/s40337-021-00418-4
93. Mahmoud MA, Daboos M, Gouda S, Othman A, Abdelmaboud M, Hussein ME, Akl M. Telemedicine (virtual clinic) effectively delivers the required healthcare service for pediatric ambulatory surgical patients during the current era of COVID-19 pandemic: A mixed descriptive study. J Pediatr Surg. (2022) 57:630-636. doi:10.1016/j.jpedsurg.2021.11.018
94. Walijee H, Sood S, Markey A, Krishnan M, Lee A, De S. Is nurse-led telephone follow-up for post-operative obstructive sleep apnoea patients effective? A prospective observational study at a paediatric tertiary centre. Int J Pediatr Otorhinolaryngol. (2020) 129:1-5. doi:10.1016/j.ijporl.2019.109766
95. Brewster RCL, Zhang J, Stewart M, Kaur R, Arellano M, Bourgeois F. A Prescription for Internet: Feasibility of a Tablet Loaner Program to Address Digital Health Inequities. Appl Clin Inform. (2023) 14(2):273-278. doi:10.1055/a-2016-7417
96. Sultan AA, Acuna AJ, Samuel LT, Rabin JM, Grits D, Gurd DP, Kuivila TE, Goodwin RC. Utilization of Telemedicine Virtual Visits in Pediatric Spinal Deformity Patients: A Comparison of Feasibility and Patient Satisfaction at a Large Academic Center. J Pediatr Orthop. (2020) 40(8):e712-e715. doi:10.1097/BPO.0000000000001553
97. Yen S. Ranney ML, Krek M, Peters JR, Mereish E, Tezanos KM, Kahler CW, Solomon J, Beard C, Spirito A. Skills to enhance positivity in suicidal adolescents: Results from a pilot randomized clinical trial. J Posit Psychol. (2020) 15(3):348-361. doi:10.1080/17439760.2019.1615105
98. Phillips D, Matheson L, Pain T, Kingston GA. Evaluation of an occupational therapy led Paediatric Burns Telehealth Review Clinic: exploring the experience of family/carers and clinicians. Rural Remote Health. (2022) 22(1):1-8. doi:10.22605/RRH6887
99. Mimila NA, Chung PJ, Elliott MN, Bethell CD, Chacon S, Biely C, Contreras S, Chavis T, Bruno Y, Moss T, Coker TR. Well-Child Care Redesign: A Mixed Methods Analysis of Parent Experiences in the PARENT Trial. Acad Pediatr. (2017) 17(7):747-754. doi:10.1016/j.acap.2017.02.004

100. Chan EYH, Liu MS, Or PC, Ma ALT. Outcomes and perception of cloud-based remote patient monitoring in children receiving automated peritoneal dialysis: a prospective study. Pediatr Nephrol. (2022) 38:2171-2178. doi:10.1007/s00467-022-05828-3

1. Dempsey
2. McLardie-Hore FE, McLachlan HL, Shafiei T, Forster DA. (2020). Proactive telephone-based peer support for breastfeeding: a cross-sectional survey of women's experiences of receiving support in the RUBY randomised controlled trial. BMJ Open. (2020) 10:1-11. doi:10.1136/bmjopen-202-040412
3. Finnegan R, Flynn A, Flanagan O. Exploring parental experiences of virtual paediatric neurodevelopmental consultations. Ir J Med Sci. (2022) 191(2):807-808. doi:10.1007/s11845-021-02583-6
4. Bell S, Karamchandani U, Malcolmson K, Moosajee M. Acceptability of telegenetics for families with genetic eye diseases. Genes. (2021) 12(2):1-9. doi:10.3390/genes12020276
5. Charnell AM, Hannon E, Burke D, Iredale MR, Sutcliffe JR. Virtual consultations: delivering outpatient clinics in paediatric surgery during the COVID-19 pandemic. Ann Pediatr Surg. (2020) 16(1):1-5. doi:10.1186/s43159-020-00060-w
6. Carretier E, Bastide M, Lachal J, Moro MR. Evaluation of the rapid implementation of telehealth during the COVID-19 pandemic: a qualitative study among adolescents and their parents. Eur Child Adolesc Psychiatry. (2022) 32:963-973. doi:10.1007/s00787-022-02108-
7. Joseph HB, Kuppusamy S, Mahalik SK, Shetty AP, Das K. Telemedicine – a boon to parents of children with health care needs during COVID-19 pandemic: A qualitative study from India. Turk Arch Pediatr. (2022) 57(5):526-531. doi:10.5152/TurkArchPediatr.2022.22046
8. Edwards LM, Parry M. (2022). Telephone consultations to manage paediatric outpatient clinics during the COVID-19 pandemic: a service evaluation. Ir J Med Sci. (2022) 191(3):977-983. doi:10.1007/s11845-021-02672-6
9. Hale AE, Bujoreanu S, LaVigne TW, Coakley R. Rapid mobilization of an evidence-based psychological intervention for pediatric pain during COVID-19: The development and deployment of the comfort ability program virtual intervention (CAP-V). Children. (2023) 10:1-14. doi:10.3390/children10091523
10. Bullock DR, Vehe RK, Zhang L, Correll CK. Telemedicine and other care models in pediatric rheumatology: An exploratory study of parents' perceptions of barriers to care and care preferences. Pediatr Rheumatol. (2017) 15(1):1-8. doi:10.1186/s12969-017-0184-y
11. Moreno L, Peck JL. Nurse Practitioner-Led Telehealth to Improve Outpatient Pediatric Tracheostomy Management in South Texas. J Pediatr Health Care. (2020) 34(3):246-255. doi:10.1016/j.pedhc.2019.11.008
12. Mollen CJ, Henien M, Jacobs LM, Myers S. Parent Perceptions on Transfers to Pediatric Emergency Departments and the Role of Telemedicine. Pediatr Emerg Care. (2019) 35(3):180-184. doi:10.1097/pec.000000000000957
13. Heath J, Dancel R, Stephens JR. Postdischarge phone calls after pediatric hospitalization: An observational study. Hosp Pediatr. (2015) 5(5):241-248. doi:10.1542/hpeds.2014-0069
14. López Seguí F, Batlle Boada A, García García JJ, López Ulldemolins A, Achotegui Del Arco A, Adroher Mas C, García Cuyàs F. Families' degree of satisfaction with pediatric telehomecare: Interventional prospective pilot study in Catalonia. JMIR Pediatr Parent. (2020) 3(1):1-8. Doi:10.2196/17517
15. Fletcher SE, Tsang VWL. The era of virtual care: Perspectives of youth on virtual appointments in COVID-19 and beyond. Paediatr Child Health. (2021) 26(4):210-213. doi:10.1093/pch/pxaa138
16. Foster CC, Macy ML, Simon N, Stephen R, Lehnig K, Bohling K, Schinasi DA. Emergency Care Connect: Extending Pediatric Emergency Care Expertise to General Emergency Departments Through Telemedicine. Acad Pediatr. (2020) 20(5):577-584. doi:10.1016/j.acap.2020.02.028
17. Castro MJ, Rodriguez RJ, Hudson B, Weersing VR, Kipke M, Peterson BS, West AE. Delivery of cognitive behavioral therapy with diverse, underresourced youth using telehealth: Advancing equity through consumer perspectives. Evid Based Pract Child Adolesc Ment Health. (2022) 8(2):206-220. doi:10.1080/23794925.2022.2062687
